# Supplementary material for: Lamina-specific immunohistochemical signatures in the olfactory bulb of healthy, Alzheimer’s and Parkinson’s disease patients
Source: Commun Biol. 2022 Jan 24;5:88. doi: 10.1038/s42003-022-03032-5 (PMC8786934; doi:10.1038/s42003-022-03032-5)
Supplement: Supplementary file 2 — Supplementary Information [file 42003_2022_3032_MOESM2_ESM.pdf]

Supplementary Table 1: Antibodies used for multiplexed immunohistochemistry on human olfactory bulb sections

Antigen	Host	Host Ig Class	Conjugate	Supplier	Product number	Conc	RRID	Secondary Antibody	Supplier/Product number	Used for Analysis
**Round 1**										
-	-	-	-	-	-	1:100	-	DAPI	Invitrogen / D1306	Yes
Histones	Mouse	IgG2b	-	MBL	MABI0301 mix	1:200	AB_11142498	DyLight 405	Jackson / 115-475-207	Yes
Collagen IV	Rabbit	IgG	-	Abcam	ab6586	1:200	AB_305584	Alexa Fluor 430	Invitrogen / A11064	
Fibrinogen	Sheep	IgG	FITC	Millipore	AB7144F	1:20	AB_2293977	-	-	
CD31	Guinea Pig	IgG	-	Synaptic Systems	351004	1:100	AB_2620105	Alexa Fluor 546	Invitrogen / A11074	
UEA Lectin	-	-	DyLight 594	Vector Labs	DL-1067	1:200	-	-	-	
HLA-DR	Mouse	IgG1	-	DAKO	M0775	1:200	AB_2313661	Alexa Fluor 647	Invitrogen / A21240	Yes
Tomato Lectin	-	-	Biotin	Vector Labs	B-1175	1:200	-	Streptavidin- PerCP	Jackson / 016-120-084	
Iba1	Chicken	IgY	-	Synaptic Systems	234006	1:200	AB_2619949	IRDye 680LT	Li-Cor / 926-68028	Yes
SMA	Mouse	IgG2a	-	eBioscience	14-9760-82	1:100	AB_2572996	IRDye 800CW	Li-Cor / 92632351	
**Round 2**										
-	-	-	-	-	-	-	-	DAPI	Invitrogen / D1306	
Synaptophysin	Mouse	IgM	-	Millipore	MAB329	1:100	AB_94786	DY395XL	Li-Cor / CSQ-0007-1	
Tyrosine Hydroxlyase	Rabbit	IgG	-	Novus Biologicals	NB300-109	1:100	AB_10077691	Alexa Fluor 488	Invitrogen / A11034	Yes
GAD65	Mouse	IgG2a	-	Abcam	Ab26113	1:200	AB_448989	Alexa Fluor 546	Invitrogen / A21133	
GAD67	Mouse	IgG2b	-	Synaptic Systems	198211	1:200	AB_2721099	Alexa Fluor 594	Invitrogen / A21145	
Parvalbumin	Chicken	IgY	-	Synaptic Systems	195006	1:100	AB_2619887	Alexa Fluor 647	Invitrogen / A21149	
Tomato Lectin	-	-	Biotin	Vector Labs	B-1175	1:200	-	Streptavidin- PerCP	Jackson / 016-120-084	Yes
NeuN	Guinea Pig		-	Millipore	ABN90P	1:200	AB_2341095	IRDye 680LT	Li-Cor / 926-6830	
Beta Amyloid [6E10]	Mouse	IgG1	-	BioLegend	803004	1:100	AB_2715854	IRDye 800CW	Li-Cor / 926-32350	Yes
**Round 3**										
-	-	-	-	-	-	-	-	DAPI	Invitrogen / D1306	
GAD67	Mouse	IgG3	-	Santa Cruz	Sc-28376	1:100	AB_627650	DY395XL	Li-Cor / CSQ-0007-3	
NeuN	Guinea Pig	IgG	-	Millipore	ABN90P	1:200	AB_2341095	Alexa Fluor 488	Invitrogen / A11073	
Neurogranin	Rabbit	IgG	-	Sigma	HPA038171	1:100	AB_2675878	Alexa Fluor 546	Invitrogen / A11035	
Parvalbumin	Sheep	IgG	-	R&D Systems	AF5058	1:100	AB_2173907	Alexa Fluor 594	Invitrogen / A11016	
Calbindin	Mouse	IgG2a	-	Abcam	Ab75524	1:200	AB_1310017	Alexa Fluor 647	Invitrogen / A21241	Yes
Tomato Lectin	-	-	Biotin	Vector Labs	B-1175	1:200	-	Streptavidin- PerCP	Jackson / 016-120-084	
Calretinin	Chicken	IgY	-	Synaptic Systems	214106	1:200	AB_2619909	IRDye 680LT	Li-Cor / 926-68028	
Beta-Amyloid [6F3D]	Mouse	IgG1	-	DAKO	M0872	1:100	AB_2056966	IRDye 800CW	Li-Cor / 926-32350	
**Round 4**										
-	-	-	-	-	-	-	-	DAPI	Invitrogen / D1306	
Tau [D-8]	Mouse	IgG2b	-	Santa Cruz	sc166060	1:100	AB_2266085	DY395XL	Li-Cor / CSQ-0007-2b	
PGP9.5	Mouse	IgG2a	-	Abcam	ab8189	1:200	AB_306343	Alexa Fluor 488	Invitrogen / A21131	Yes
Parvalbumin	Mouse	IgG1	-	Millipore	MAB1572	1:200	AB_2174013	Alexa Fluor 546	Invitrogen / A21123	
Calbindin	Chicken	IgY	-	Synaptic Systems	214 006	1:200	AB_2619903	Alexa Fluor 594	Invitrogen / A11042	
Calretinin	Guinea Pig	IgG	-	Synaptic Systems	214 104	1:200	AB_10635160	Alexa Fluor 647	Invitrogen / A21450	

Tomato Lectin	-	-	Biotin	Vector Labs	B-1175	1:200	-	Streptavidin- PerCP	Jackson / 016-120-084	
Calretinin	Rabbit	IgG	-	Millipore	AB5054	1:200	AB_2068506	IRDye 680LT	Li-Cor / 926-68023	Yes
PSA-NCAM	Mouse	IgM	-	eBiosceince	14-9118-82	1:100	AB_2572932	IRDye 800CW	Li-Cor / 926-32280	
**Round 5**										
-	-	-	-	-	-	-	-	DAPI	Invitrogen / D1306	
Glutaminase	Rabbit	IgG	-	Novus	NBP2-29940	1:100		Alexa Fluor 430	Invitrogen / A11064	
MAP2	Mouse	IgG3	-	R&D systems	MAB8304	1:100	AB_2814693	Alexa Fluor 488	Invitrogen / A21151	
VGLUT2	Guinea Pig	IgG	-	Millipore	AB2251	1:200	AB_1587626	Alexa Fluor 546	Invitrogen / A11074	
phosphoTau pSer202 [AT8]	Mouse	IgG1	-	Invitrogen	MN1020	1:100	AB_223647	Alexa Fluor 594	Invitrogen / A21125	Yes
Alpha synuclein [p-syn/81A]	Mouse	IgG2a	-	Abcam	ab184674	1:200	AB_2819037	Alexa Fluor 647	Invitrogen / A21241	Yes
Tomato Lectin	-	-	Biotin	Vector Labs	B-1175	1:200	-	Streptavidin- PerCP	Jackson / 016-120-084	
NeuN	Chicken	IgY	-	Millipore	ABN91	1:200	AB_11205760	IRDye 680LT	Li-Cor / 926-68028	Yes
Beta Amyloid [MOAB-2]	Mouse	IgG2b	-	Novus	NBP2-13075	1:200		IRDye 800CW	Li-Cor / 926-32352	
**Round 6**										
-	-	-	-	-	-	-	-	DAPI	Invitrogen / D1306	
NCAM	Rabbit	IgG	-	Millipore	AB5032	1:100	AB_2291692	Alexa Fluor 430	Invitrogen / A11064	
MAP2	Mouse	IgG3	-	R&D systems	MAB8304	1:100	AB_2814693	Alexa Fluor 488	Invitrogen / A21151	Yes
Neurofilament Light [NFL2/3 mix]	Mouse	IgG1	-	BioLegend	846002 + 845902	1:200	AB_2566775, AB_2571916	Alexa Fluor 546	Invitrogen / A21123	Yes
Neurofilament Medium	Mouse	IgG2a	-	Novus	NB500-446	1:200	AB_10003004	Alexa Fluor 594	Invitrogen / A21135	
Neurofilament Heavy	Chicken	IgY	-	Millipore	AB5539	1:200	AB_11212161	Alexa Fluor 647	Invitrogen / A21449	Yes
Tomato Lectin	-	-	Biotin	Vector Labs	B-1175	1:200	-	Streptavidin- PerCP	Jackson / 016-120-084	
Tubulin β3	Mouse	IgG2b	-	Sigma	T-8660	1:200	AB_477590	IRDye 680LT	Li-Cor / 926-68052	
Synaptophysin	Mouse	IgM	-	Millipore	MAB329	1:200	AB_94786	IRDye 800CW	Li-Cor / 926-32280	Yes
**Round 7**										
-	-	-	-	-	-	-	-	DAPI	Invitrogen / D1306	
Myelin Basic Protein	Guinea Pig	IgG	-	Synaptic Systems	295004	1:200	AB_2620037	DY395XL	Li-Cor / CSQ-0007-0	
CNPase	Mouse	IgG2b	-	Novus	NBP2-46617	1:200		Alexa Fluor 488	Invitrogen / A21141	Yes
Olig2	Mouse	IgG2a	-	Millipore	MABN50	1:200	AB_10807410	Alexa Fluor 546	Invitrogen / A21133	
Myelin Basic Protein	Mouse	IgG1	-	Abcam	ab24567	1:200	AB_448144	Alexa Fluor 594	Invitrogen / A21125	
Myelin Basic Protein	Rat	IgG2a	-	Millipore	MAB386	1:200	AB_94975	Alexa Fluor 647	Invitrogen / A21247	Yes
Tomato Lectin	-	-	Biotin	Vector Labs	B-1175	1:200	-	Streptavidin- PerCP	Jackson / 016-120-084	
Myelin Basic Protein	Chicken	IgY	-	GeneTex	GTX85456	1:200	AB_10620404	IRDye 680LT	Li-Cor / 926-68028	
GalC	Rabbit	IgG	-	Millipore	AB142	1:100	AB_90632	IRDye 800CW	Li-Cor / 926-32213	
**Round 8**										
-	-	-	-	-	-	-	-	DAPI	Invitrogen / D1306	
GFAP [cocktail]	Mouse	IgG2b	-	BD Biosciences	556330	1:200	AB_396368	DY395XL	Li-Cor / CSQ-0007-2b	
GFAP	Rabbit	IgG	-	DAKO	Z0334	1:200	AB_10013382	Alexa Fluor 488	Invitrogen / A21134	Yes
Sox2	Rat	IgG2a	-	eBioscience	14-9811-82	1:200	AB_11219471	Alexa Fluor 546	Invitrogen / A11081	
GFAP	Chicken	IgY	-	ThermoFisher	PA1-10004	1:200	AB_1074620	Alexa Fluor 594	Invitrogen / A11042	
S100	Mouse	IgG2a	-	Millipore	MAB079-1	1:200	AB_571112	Alexa Fluor 647	Invitrogen / A21241	Yes

Tomato Lectin	-	-	Biotin	Vector Labs	B-1175	1:200	-	Streptavidin- PerCP	Jackson / 016-120-084	
EAAT1	Guinea Pig	IgG	-	Synaptic Systems	250114	1:200	AB_2619957	IRDye 680LT	Li-Cor / 926-6830	
Aquaporin 4	Mouse	IgG3	-	Abcam	ab9512	1:200	AB_307299	IRDye 800CW	Li-Cor / RAS7230953	
**Round 9**										
-	-	-	-	-	-	-	-	DAPI	Invitrogen / D1306	
Vimentin	Chicken	IgY	-	Millipore	AB5733	1:100	AB_11212377	DY395XL	Li-Cor / CSQ-00071-CK	
Fibrinogen	Sheep	IgG	FITC	Millipore	AB7144F	1:20	AB_2293977	-	-	
Collagen IV	Rabbit	IgG		Abcam	ab6586	1:100	AB_305584	Alexa Fluor 546	Invitrogen / A11035	Yes
UEA Lectin	-	-	DyLight 594	Vector Labs	DL-1067	1:100	-	-	-	Yes
CD31	Guinea Pig	IgG	-	Synaptic Systems	351004	1:100	AB_2620105	Alexa Fluor 647	Invitrogen / A21450	Yes
Tomato Lectin	-	-	Biotin	Vector Labs	B-1175	1:200	-	Streptavidin- PerCP	Jackson / 016-120-084	
Nestin	Mouse	IgG1	-	Millipore	MAB5326	1:100	AB_2251134	IRDye 680LT	Li-Cor / 926-68050	
Smooth Muscle Actin	Mouse	IgG2a	-	eBioscience	14-9760-82	1:100	AB_2572996	IRDye 800CW	Li-Cor / 926-32351	
**Round 10**										
-	-	-	-	-	-	-	-	DAPI	Invitrogen / D1306	
5T4	Rabbit	IgG	-	Novus	NBP1-90315	1:100	AB_11038774	Alexa Fluor 430	Invitrogen / A11064	
MAP2	Mouse	IgG3	-	R&D systems	MAB8304	1:100	AB_2814693	Alexa Fluor 488	Invitrogen / A21151	
OMP	Mouse	IgG2a	-	Santa Cruz	sc-365818	1:100	AB_10842164	Alexa Fluor 546	Invitrogen / A21133	Yes
phosphoTau pSer202 [AT8]	Mouse	IgG1	-	Invitrogen	MN1020	1:100	AB_223647	Alexa Fluor 594	Invitrogen / A21125	
Beta Amyloid [MOAB-2]	Mouse	IgG2b	-	Novus	NBP2-13075	1:100		Alexa Fluor 647	Invitrogen / A21242	
Tomato Lectin	-	-	Biotin	Vector Labs	B-1175	1:200	-	Streptavidin- PerCP	Jackson / 016-120-084	
NeuN	Guinea Pig	IgG	-	Millipore	ABN90P	1:200	AB_2341095	IRDye 680LT	Li-Cor / 926-6830	
Tyrosine Hydroxylase	Chicken	IgY	-	Abcam	ab76442	1:100	AB_1524535	IRDye 800CW	Li-Cor / 926-32218	

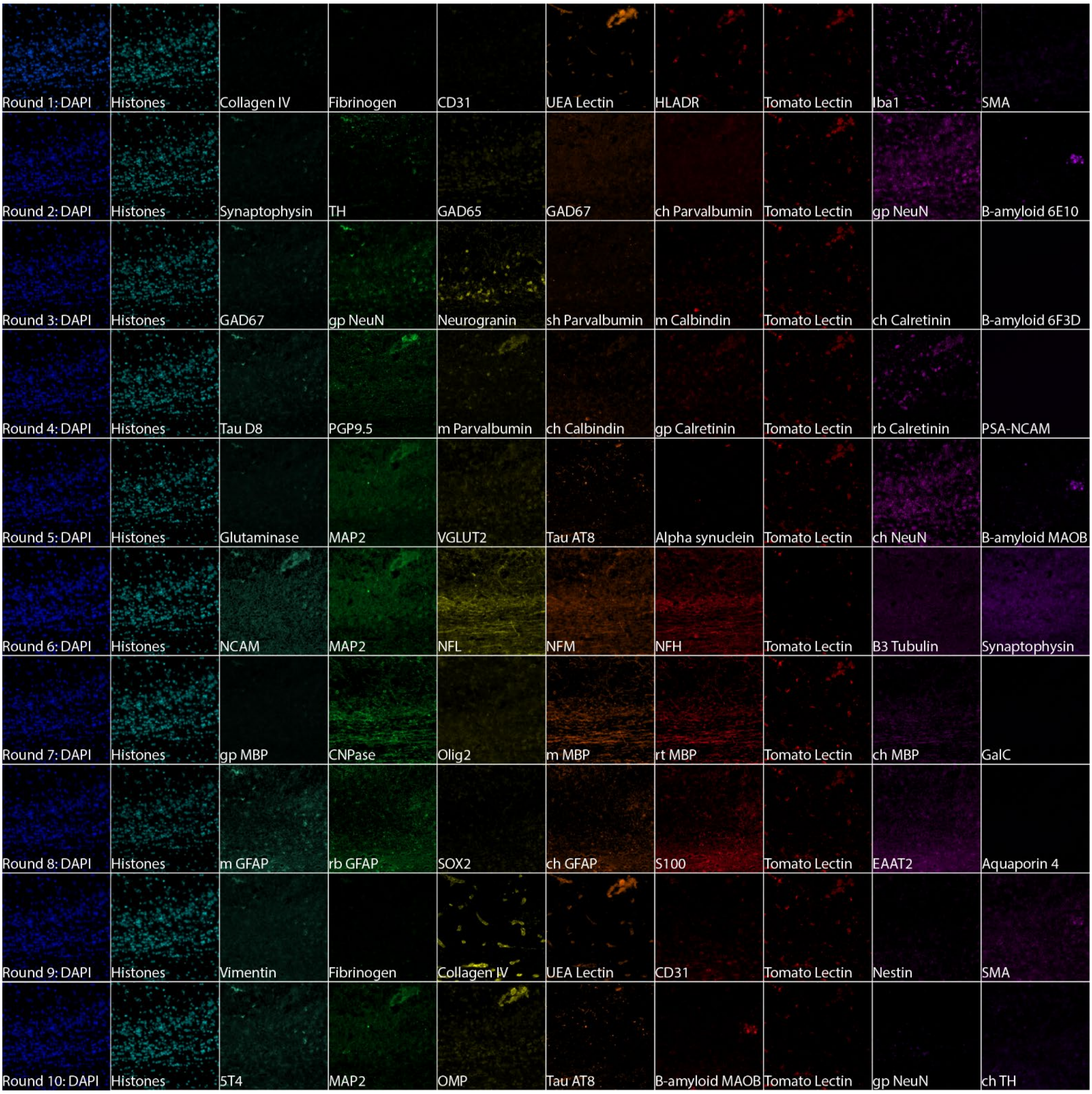

Supplementary Figure 1. Summary of antibody labelling and empirical validation of MP-IHC labelling. Example labelling of all antibodies included in this study on case AZ90. The labelling was empirically validated based on the unique design of the antibody panels, whereby, each antibody used in each labelling round predominantly targeted a spatially distinct sub-cellular location or tissue structure, with each primary antibody selected from a different host species and/or immunoglobulin class/subclass. The primary antibodies were visualised using an appropriate secondary antibody conjugated to a different spectrally non-overlapping fluorophore, and the resulting fluorescence signal from each antibody was imaged in a different spectrally non-overlapping channel with minimal spectral crosstalk as previously reported (Maric et al., 2021). The results show no significant cross-reactivity between antibodies, nor spectral crosstalk between fluorescence signals in each imaging channel for each labelling round.

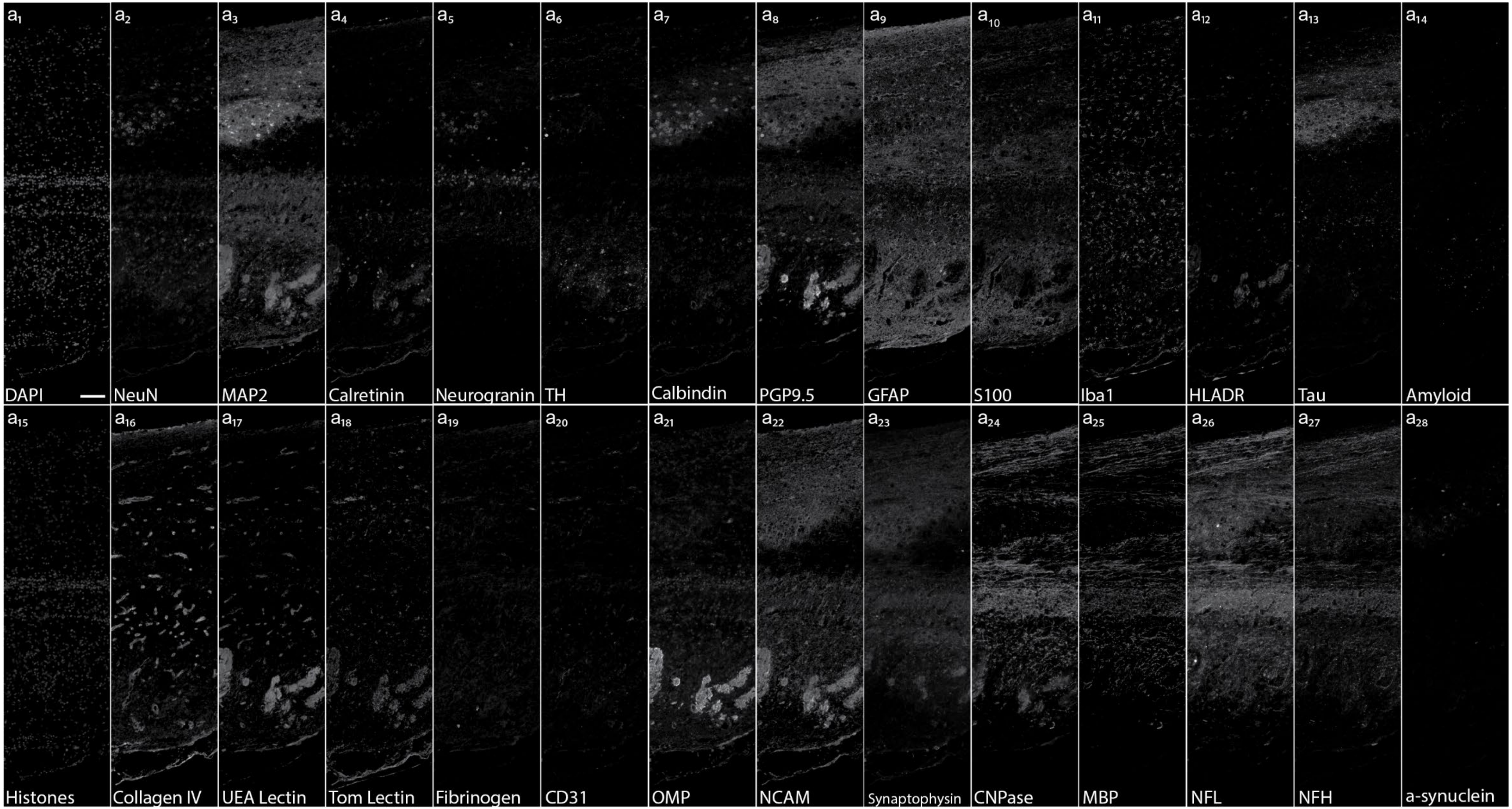

Supplementary Figure 2. Summary of neurochemical markers in the OB of AZ90. **a** A central region from a sagittal AD human OB section, showing immunoreactivity for markers of neuronal subtypes (**a**$_{2\text{-}8}$), glia (**a**$_{9\text{-}12}$), pathological aggregates (**a**$_{13\text{-}14,\,28}$), blood vessel components (**a**$_{16\text{-}20}$), glomeruli (**a**$_{21\text{-}23}$) and axons (**a**$_{24\text{-}27}$) across all layers.

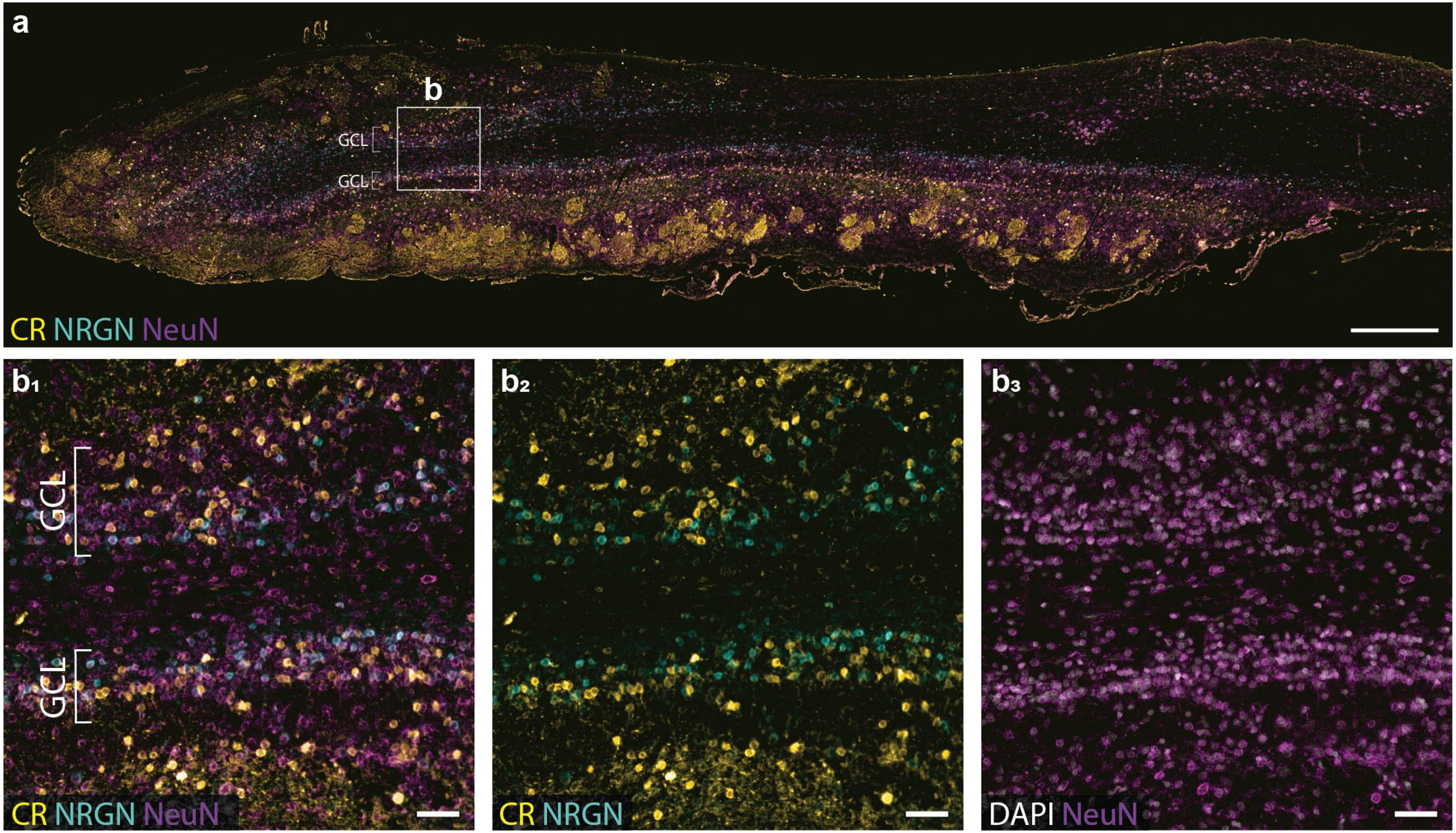

Supplementary Figure 3: Granule cell diversity in the human OB. a Overview of calretinin, neurogranin and NeuN labelling in a human AD OB section. **b** Higher power image of the granule cell layer showing that calretinin (CR) and neurogranin (NRGN) label two non-overlapping populations of granule cells. Another subset of granule cells label with NeuN but not calretinin or neurogranin. Scale bars 500 µm (a), 50 µm (b).

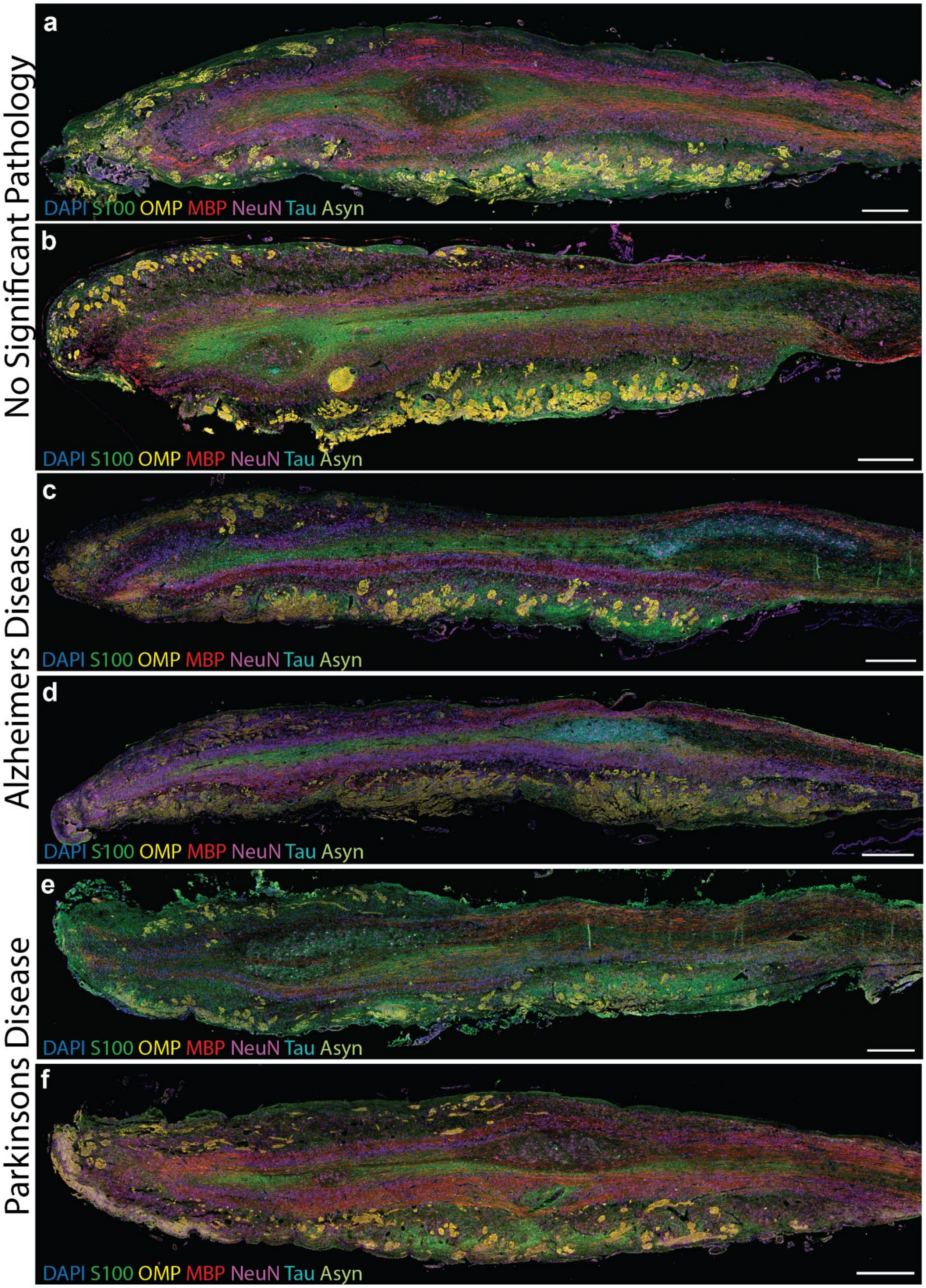

Supplementary Figure 4. Comparison of cortical layers in AD, PD and NSP cases. Using multiplex IHC the OB layers are clearly identified in NSPl (**a, b**), AD (**c, d**) and PD (**e, f**) cases, despite considerable variability in shape and structure between individuals and disease groups. Qualitatively, the white matter tract labelled with MBP (red) is thinner in AD and PD than in NSP cases. Glomeruli labelled with OMP (yellow) appear to be smaller and less dense in the PD bulbs. Pathological aggregates of tau and α-synuclein are concentrated with the AON of AD and PD cases, respectively. Scale bars 500 µm.

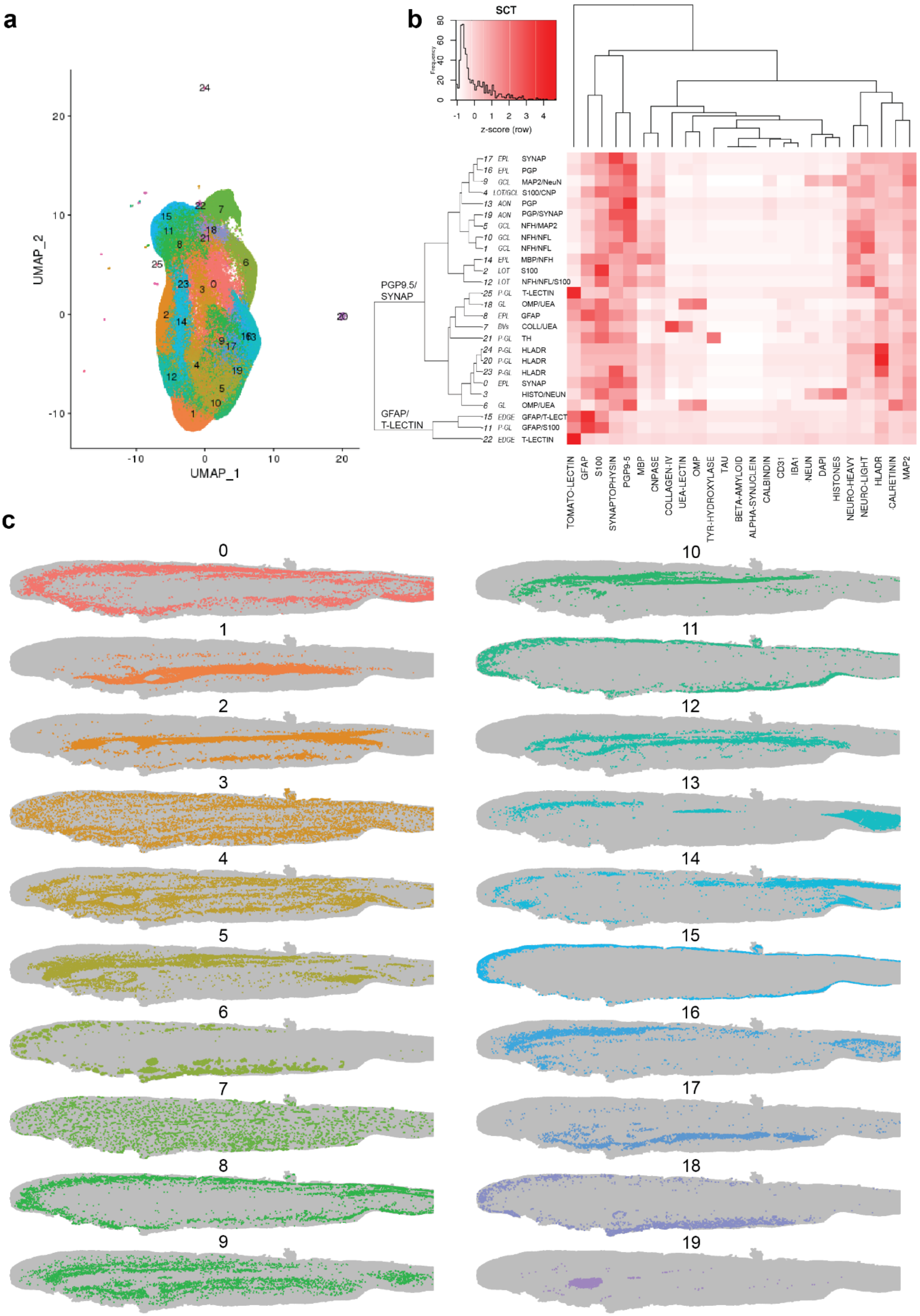

a
UMAP_2
UMAP_1
b
SCT
Frequency
z-score (row)
PGP9.5/
SYNAP
GFAP/
T-LECTIN
17 EPL SYNAP
16 EPL PGP
9 GCL MAP2/NeuN
4 LOT/GCL S100/CNP
13 AON PGP
19 AON PGP/SYNAP
5 GCL NFH/MAP2
10 GCL NFH/NFL
1 GCL NFH/NFL
14 EPL MBP/NFH
2 LOT S100
12 LOT NFH/NFL/S100
25 P-GL T-LECTIN
18 GL OMP/UEA
8 EPL GFAP
7 BVs COLL/UEA
21 P-GL TH
24 P-GL HLADR
20 P-GL HLADR
23 P-GL HLADR
0 EPL SYNAP
3 HISTO/NEUN
6 GL OMP/UEA
15 EDGE GFAP/T-LECT
11 P-GL GFAP/S100
22 EDGE T-LECTIN
TOMATO-LECTIN
GFAP
S100
SYNAPTOPHYSIN
PGP9-5
MBP
CNPASE
COLLAGEN-IV
UEA-LECTIN
OMP
TYR-HYDROXYLASE
TAU
BETA-AMYLOID
ALPHA-SYNUCLEIN
CALBINDIN
CD31
IBA1
NEUN
DAPI
HISTONES
NEURO-HEAVY
NEURO-LIGHT
HLADR
CALRETININ
MAP2
c
0
1
2
3
4
5
6
7
8
9
10
11
12
13
14
15
16
17
18
19

Supplementary Figure 5. Individual section cluster analysis for case H250. As per Figure 3, clustering of pixel bins for NSP case H250 corresponded to layer anatomy, illustrating the consistency of the approach. (**a)** UMAP of pixel bin data coloured by cluster. (**b)** Heat map of z-score of labelling intensity for markers within each cluster. Clusters are organised based on a hierarchical tree. Each cluster is identified by a number and a three-letter code indicating the layer it is localised to, based on visual inspection of the slide plot for that cluster, as well as the one or two of most intensely labelled or distinct markers for that cluster. (**c)** Individual slide plots of clusters 0-19 illustrating the unique spatial distribution of each cluster. Abbreviations: AON, anterior olfactory nucleus; BVs, blood vessels; EPL, external plexiform layer; GL, glomerular layer; GCL, granule cell layer; LOT, lateral olfactory tract; P-GL, peri-glomerular.

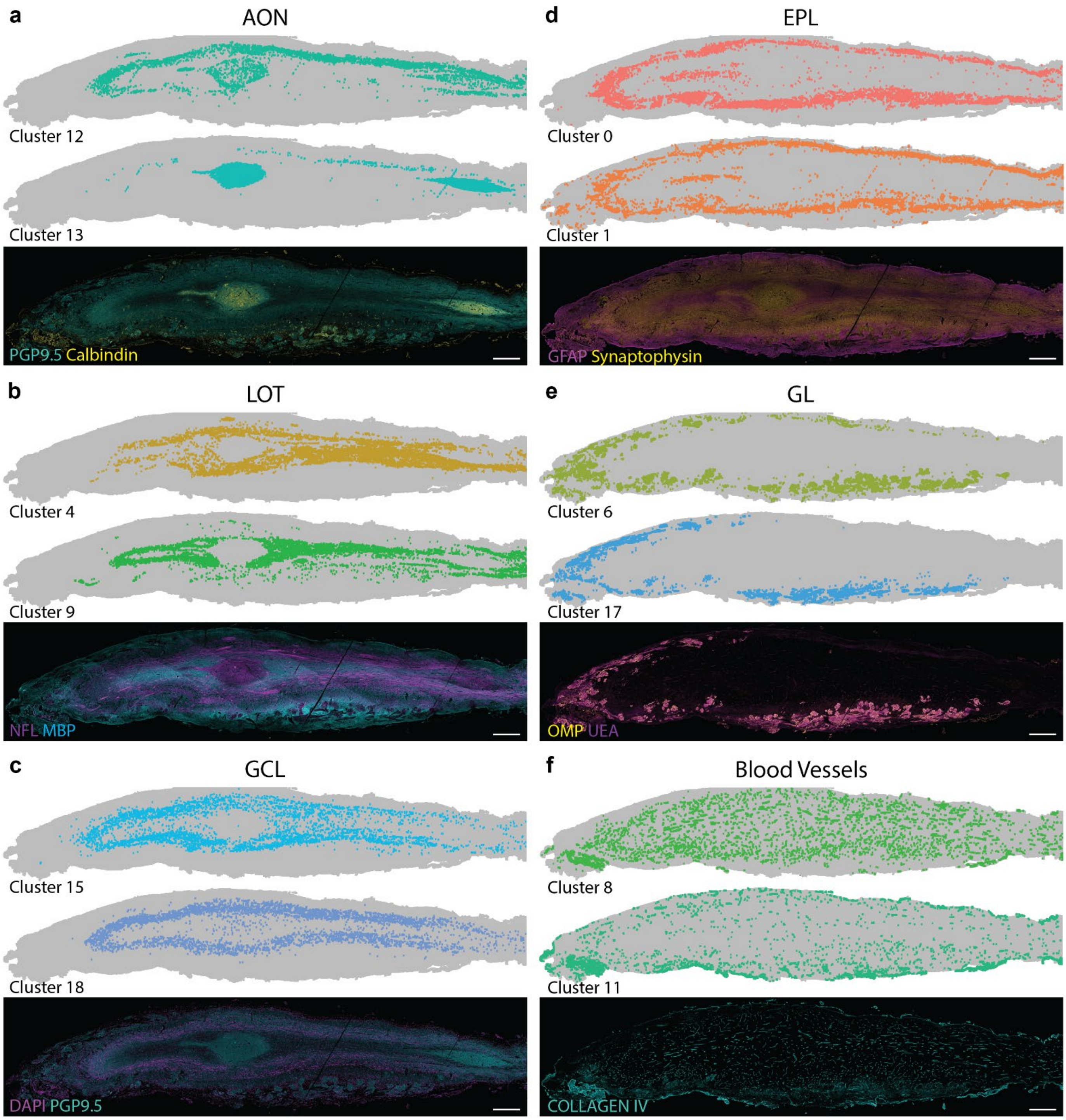

Supplementary Figure 6. Comparison of clusters and antibody labelling to identify anatomical layers in the human OB. Slide plots of clusters for NSP bulb H190 that most specifically correspond to OB layers and the antibody labelling of markers most highly represented in those clusters. (**a)** The anterior olfactory nucleus (AON) is identified by clusters 12 and 13, which show high expression of PGP9.5 and calbindin. (**b**) The lateral olfactory tract (LOT) is identified by clusters 3 and 4, showing high expression neurofilament light and myelin basic protein. (**c**) The granule cell layer (GCL) is identified by clusters 15 and 18, showing high expression of DAPI and PGP9.5. (**d**) The external plexiform layer (EPL) is identified by cluster 0 and 1, showing high expression of GFAP and synaptophysin. (**e**) The glomerular layer (GL) is identified by cluster 6 and 17 which show high OMP and UEA-I lectin expression. (**f**) Blood vessels are identified by cluster 8 and 11 and show high UEA-I lectin and collagen IV expression.

AZ84

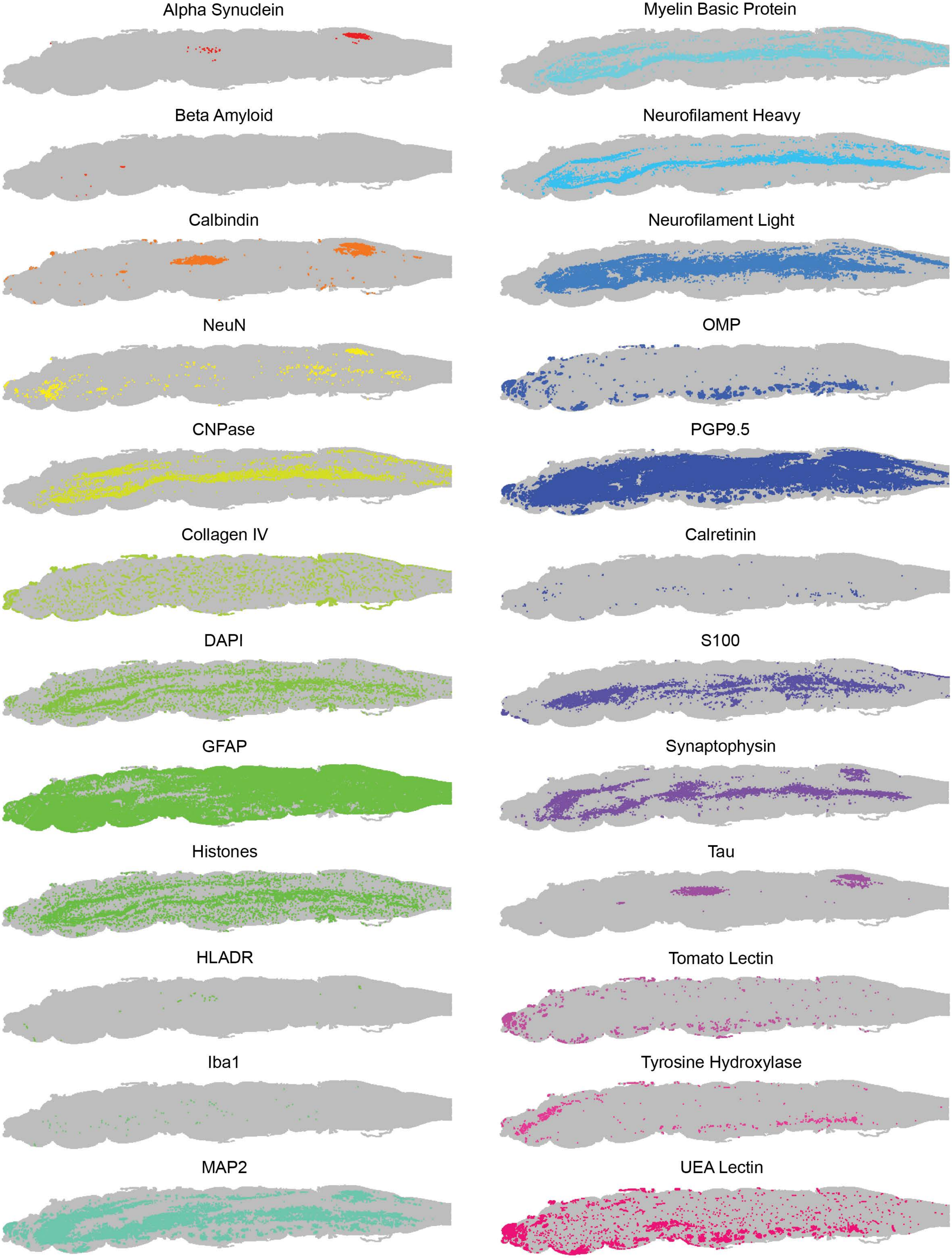
Alpha Synuclein
Myelin Basic Protein
Beta Amyloid
Neurofilament Heavy
Calbindin
Neurofilament Light
NeuN
OMP
CNPase
PGP9.5
Collagen IV
Calretinin
DAPI
S100
GFAP
Synaptophysin
Histones
Tau
HLADR
Tomato Lectin
Iba1
Tyrosine Hydroxylase
MAP2
UEA Lectin

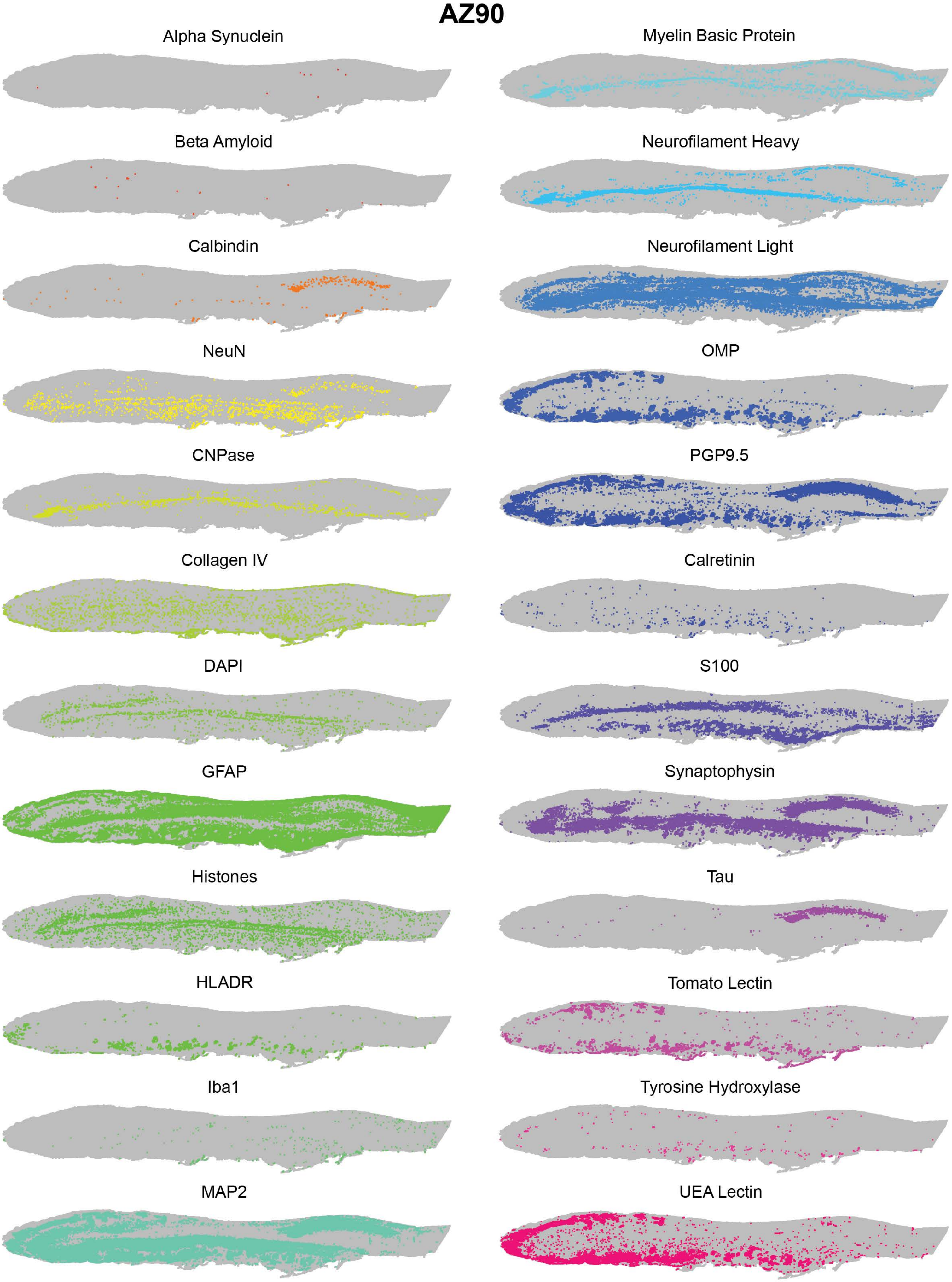
AZ90
Alpha Synuclein
Myelin Basic Protein
Beta Amyloid
Neurofilament Heavy
Calbindin
Neurofilament Light
NeuN
OMP
CNPase
PGP9.5
Collagen IV
Calretinin
DAPI
S100
GFAP
Synaptophysin
Histones
Tau
HLADR
Tomato Lectin
Iba1
Tyrosine Hydroxylase
MAP2
UEA Lectin

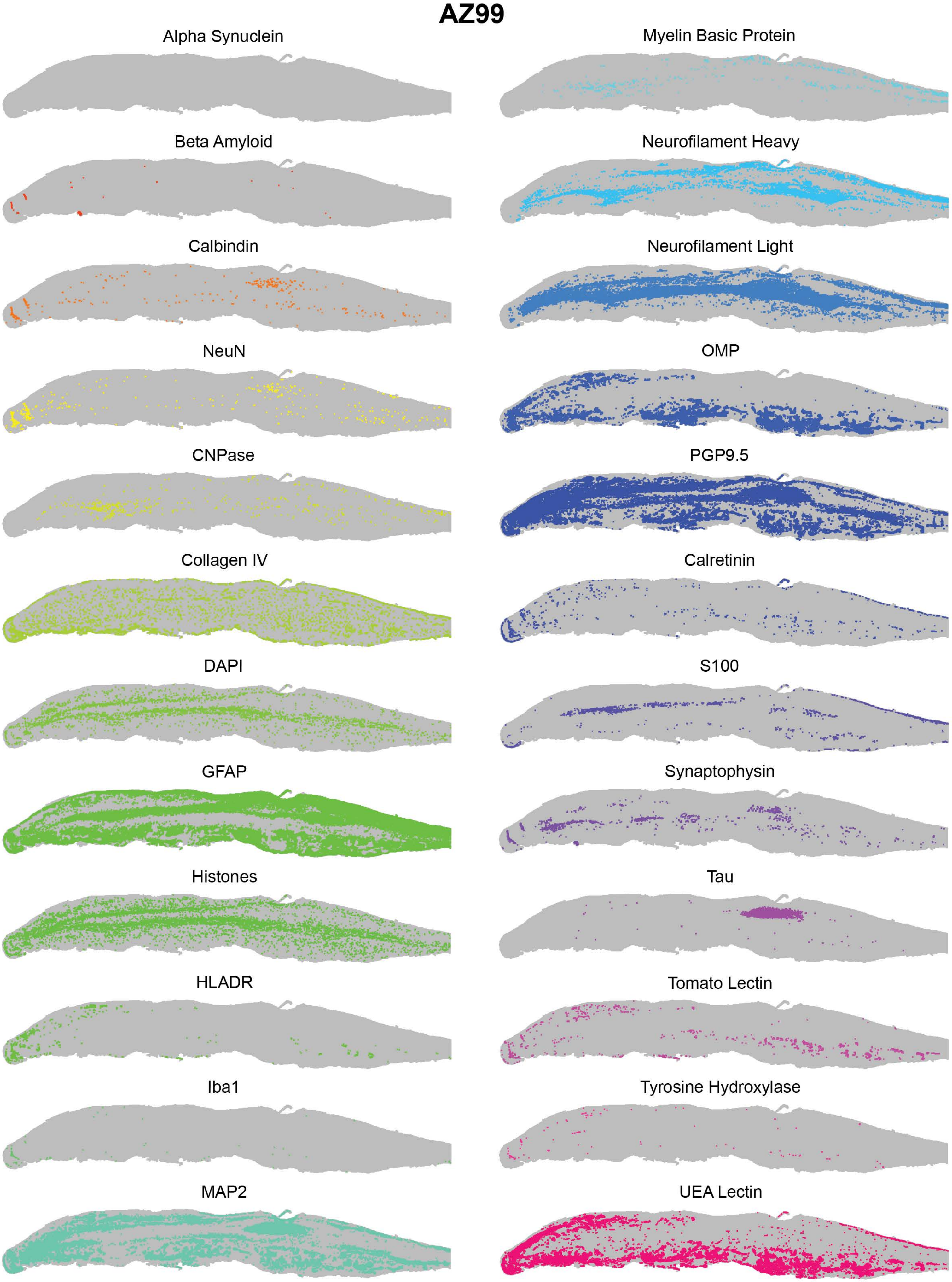
AZ99
Alpha Synuclein
Myelin Basic Protein
Beta Amyloid
Neurofilament Heavy
Calbindin
Neurofilament Light
NeuN
OMP
CNPase
PGP9.5
Collagen IV
Calretinin
DAPI
S100
GFAP
Synaptophysin
Histones
Tau
HLADR
Tomato Lectin
Iba1
Tyrosine Hydroxylase
MAP2
UEA Lectin

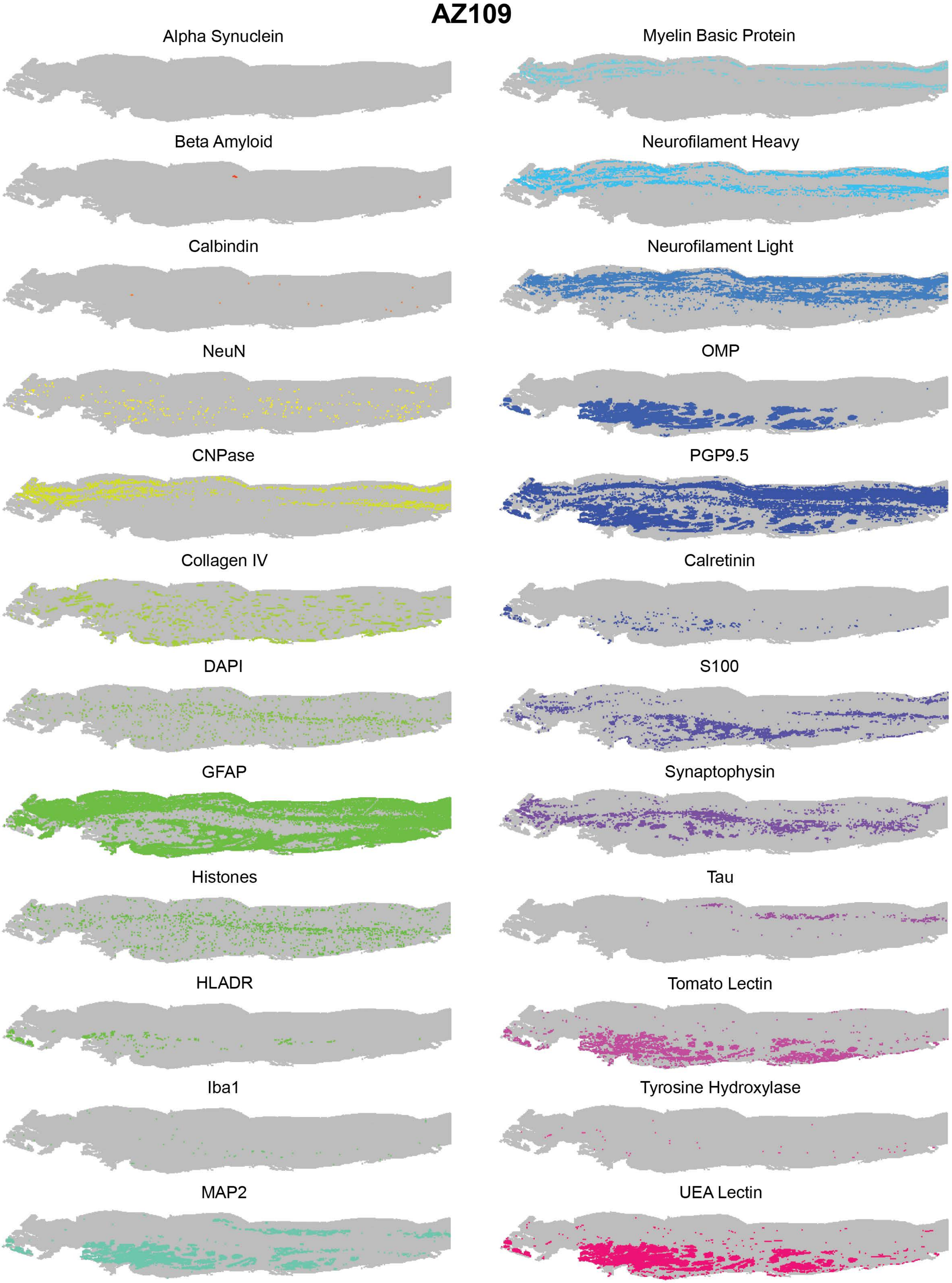
AZ109
Alpha Synuclein
Myelin Basic Protein
Beta Amyloid
Neurofilament Heavy
Calbindin
Neurofilament Light
NeuN
OMP
CNPase
PGP9.5
Collagen IV
Calretinin
DAPI
S100
GFAP
Synaptophysin
Histones
Tau
HLADR
Tomato Lectin
Iba1
Tyrosine Hydroxylase
MAP2
UEA Lectin

H251

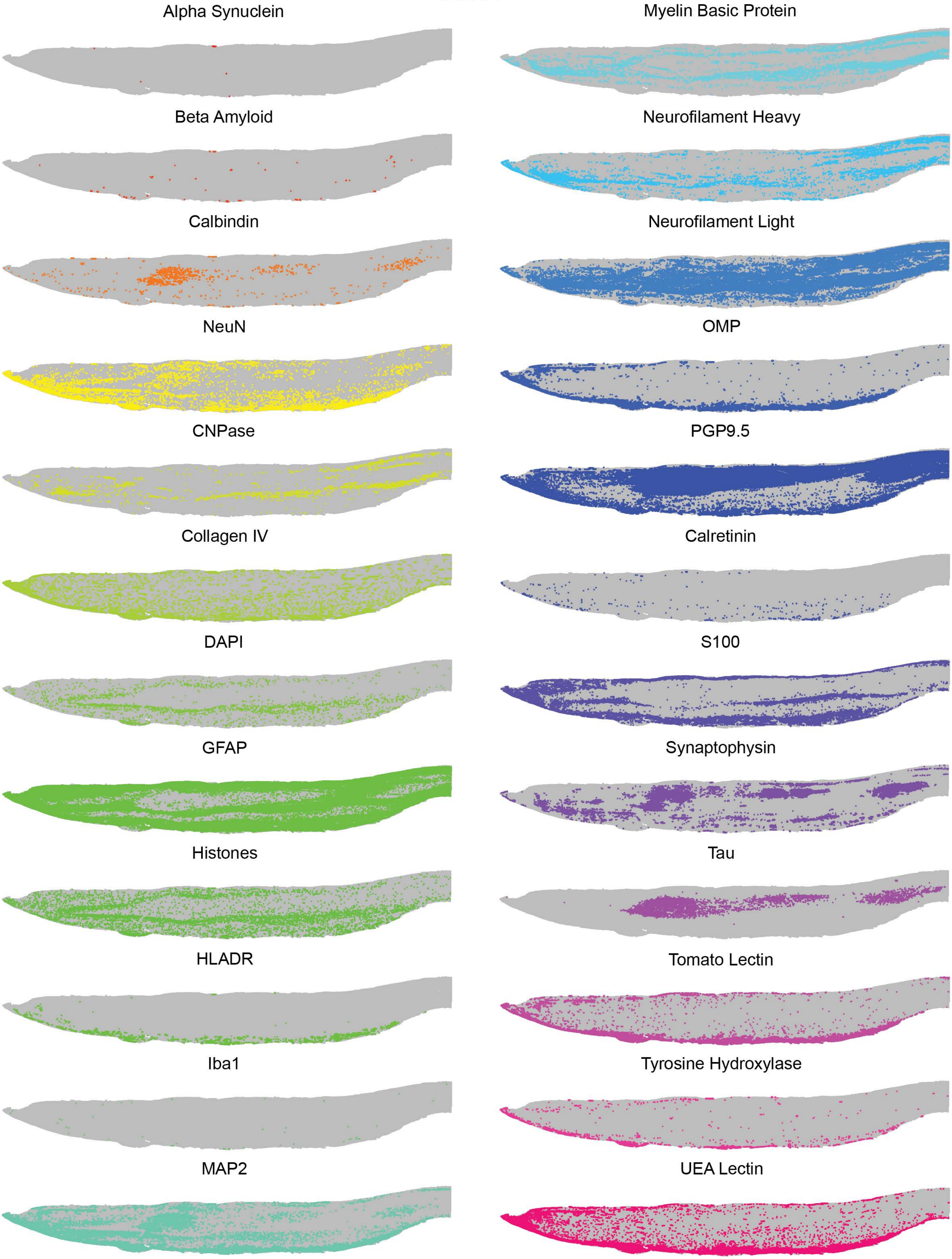
Alpha Synuclein
Myelin Basic Protein
Beta Amyloid
Neurofilament Heavy
Calbindin
Neurofilament Light
NeuN
OMP
CNPase
PGP9.5
Collagen IV
Calretinin
DAPI
S100
GFAP
Synaptophysin
Histones
Tau
HLADR
Tomato Lectin
Iba1
Tyrosine Hydroxylase
MAP2
UEA Lectin

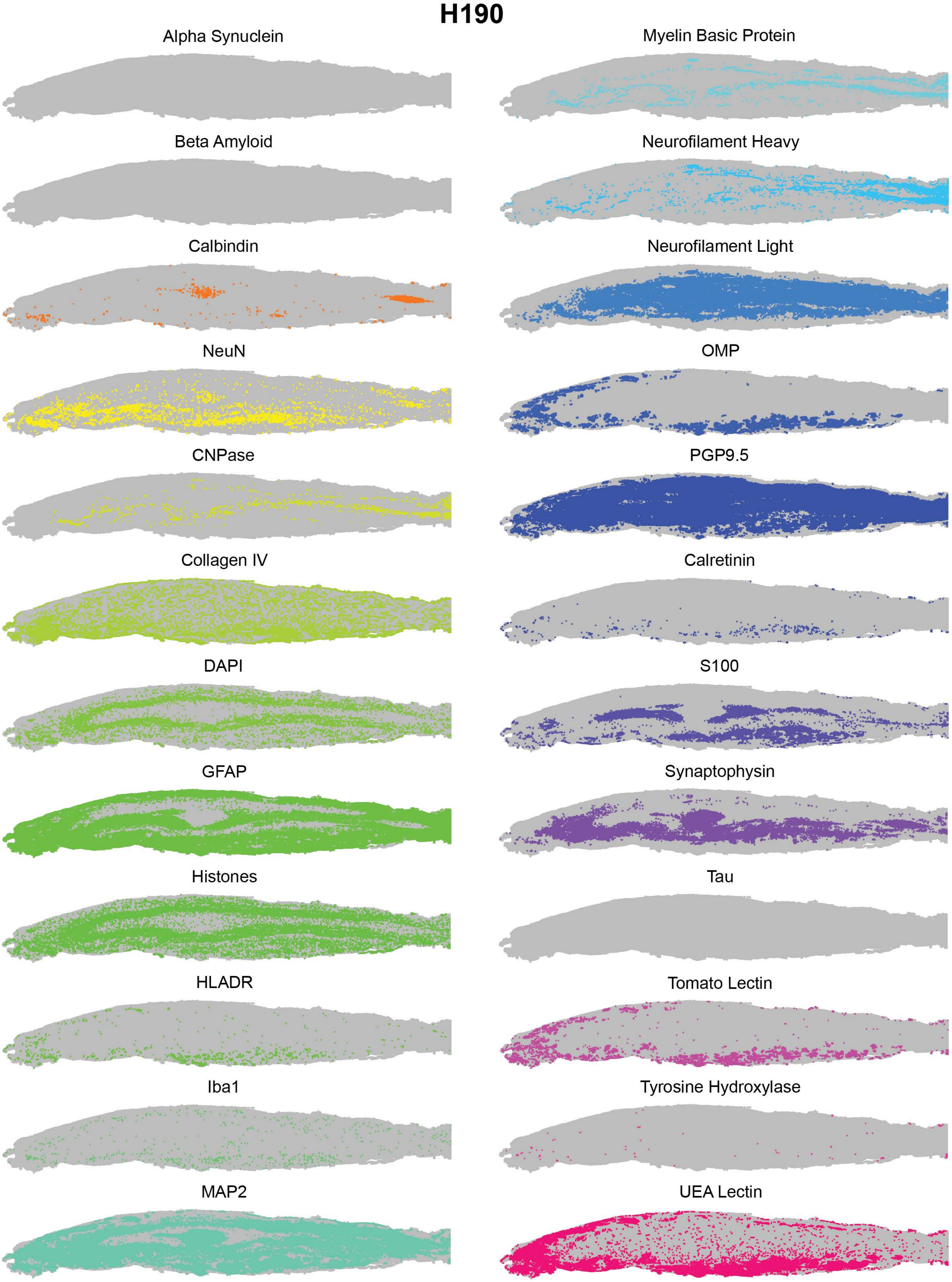
H190
Alpha Synuclein
Myelin Basic Protein
Beta Amyloid
Neurofilament Heavy
Calbindin
Neurofilament Light
NeuN
OMP
CNPase
PGP9.5
Collagen IV
Calretinin
DAPI
S100
GFAP
Synaptophysin
Histones
Tau
HLADR
Tomato Lectin
Iba1
Tyrosine Hydroxylase
MAP2
UEA Lectin

H250

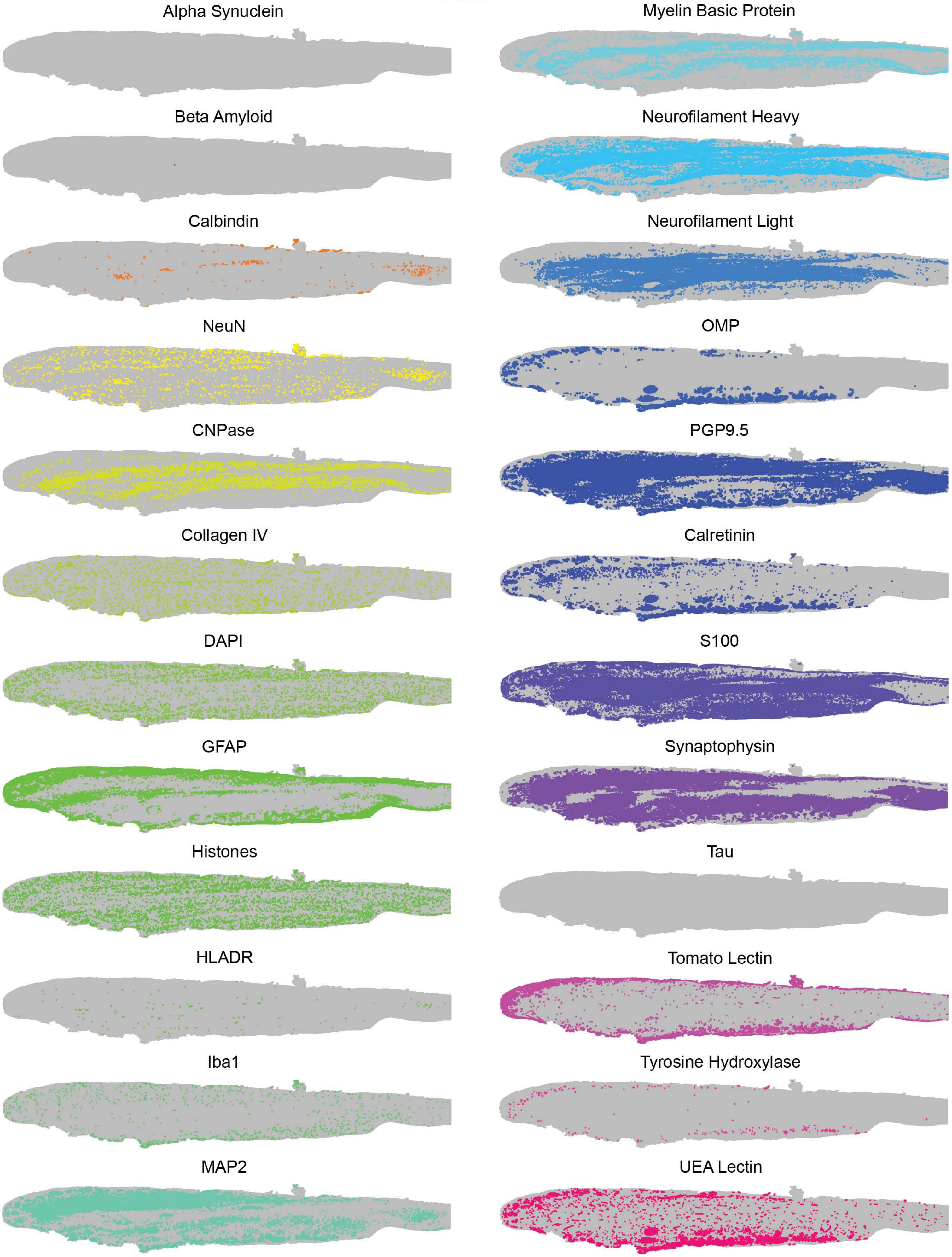
Alpha Synuclein
Myelin Basic Protein
Beta Amyloid
Neurofilament Heavy
Calbindin
Neurofilament Light
NeuN
OMP
CNPase
PGP9.5
Collagen IV
Calretinin
DAPI
S100
GFAP
Synaptophysin
Histones
Tau
HLADR
Tomato Lectin
Iba1
Tyrosine Hydroxylase
MAP2
UEA Lectin

OFB6A

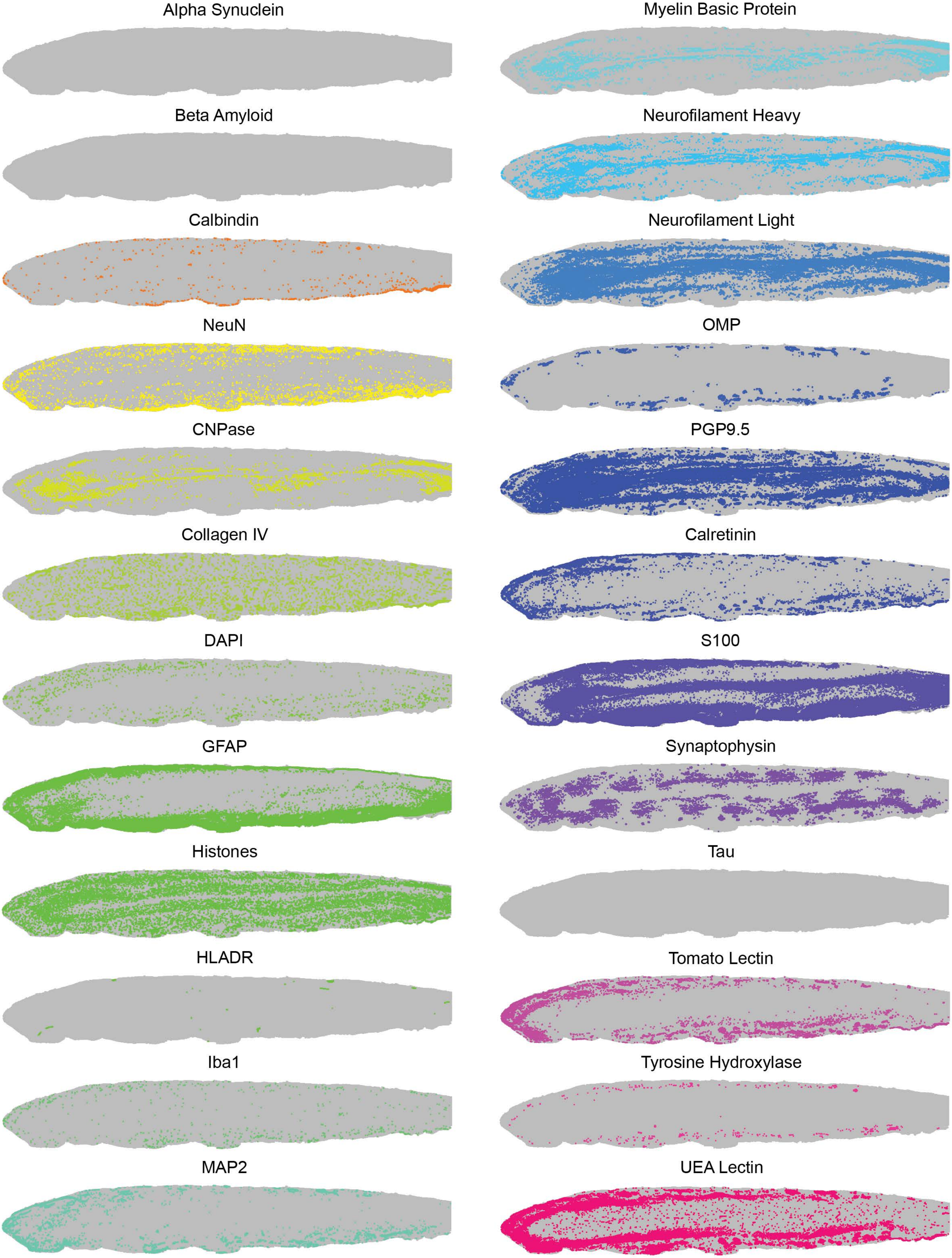
Alpha Synuclein
Myelin Basic Protein
Beta Amyloid
Neurofilament Heavy
Calbindin
Neurofilament Light
NeuN
OMP
CNPase
PGP9.5
Collagen IV
Calretinin
DAPI
S100
GFAP
Synaptophysin
Histones
Tau
HLADR
Tomato Lectin
Iba1
Tyrosine Hydroxylase
MAP2
UEA Lectin

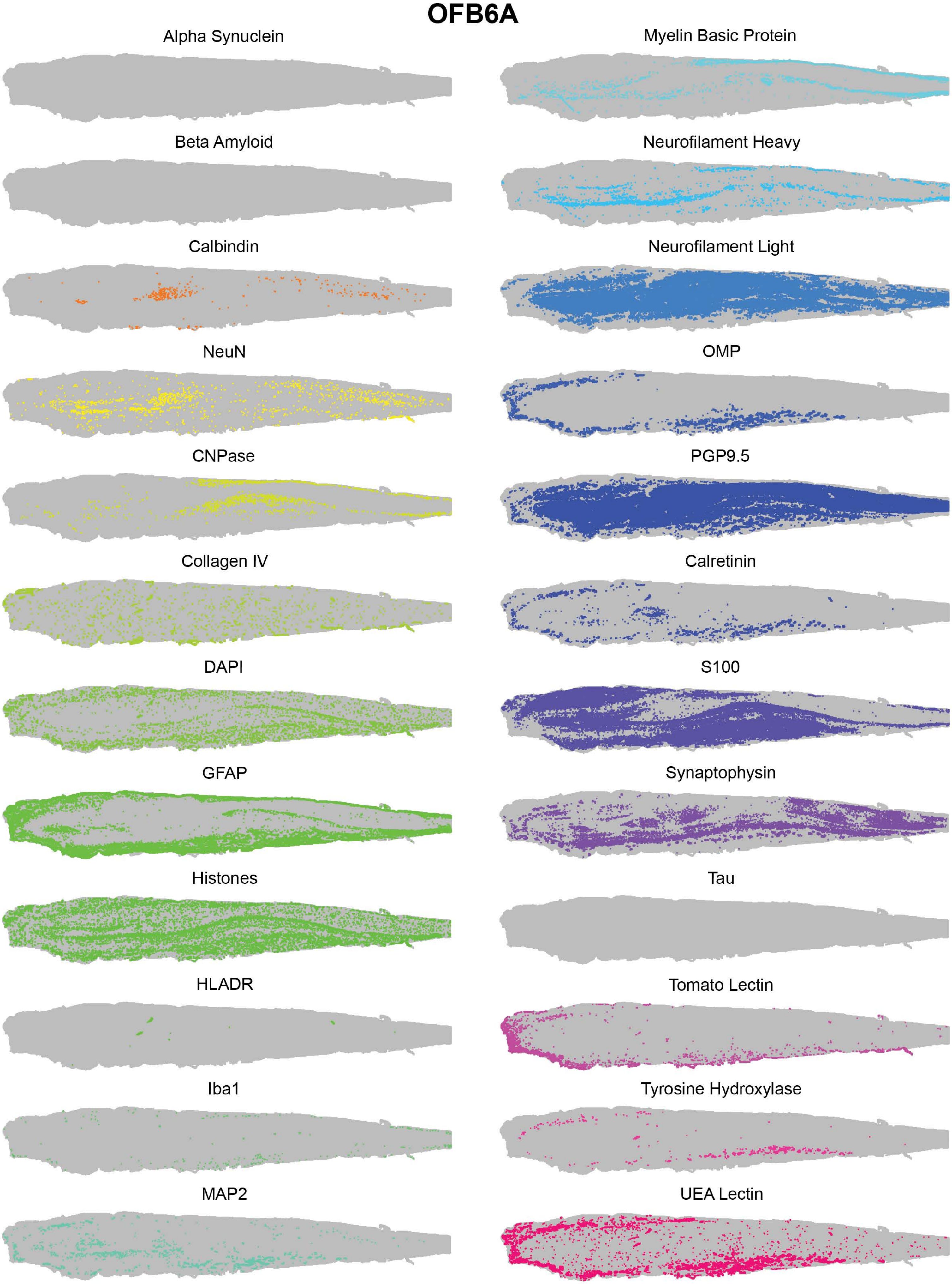
OFB6A
Alpha Synuclein
Myelin Basic Protein
Beta Amyloid
Neurofilament Heavy
Calbindin
Neurofilament Light
NeuN
OMP
CNPase
PGP9.5
Collagen IV
Calretinin
DAPI
S100
GFAP
Synaptophysin
Histones
Tau
HLADR
Tomato Lectin
Iba1
Tyrosine Hydroxylase
MAP2
UEA Lectin

PD52

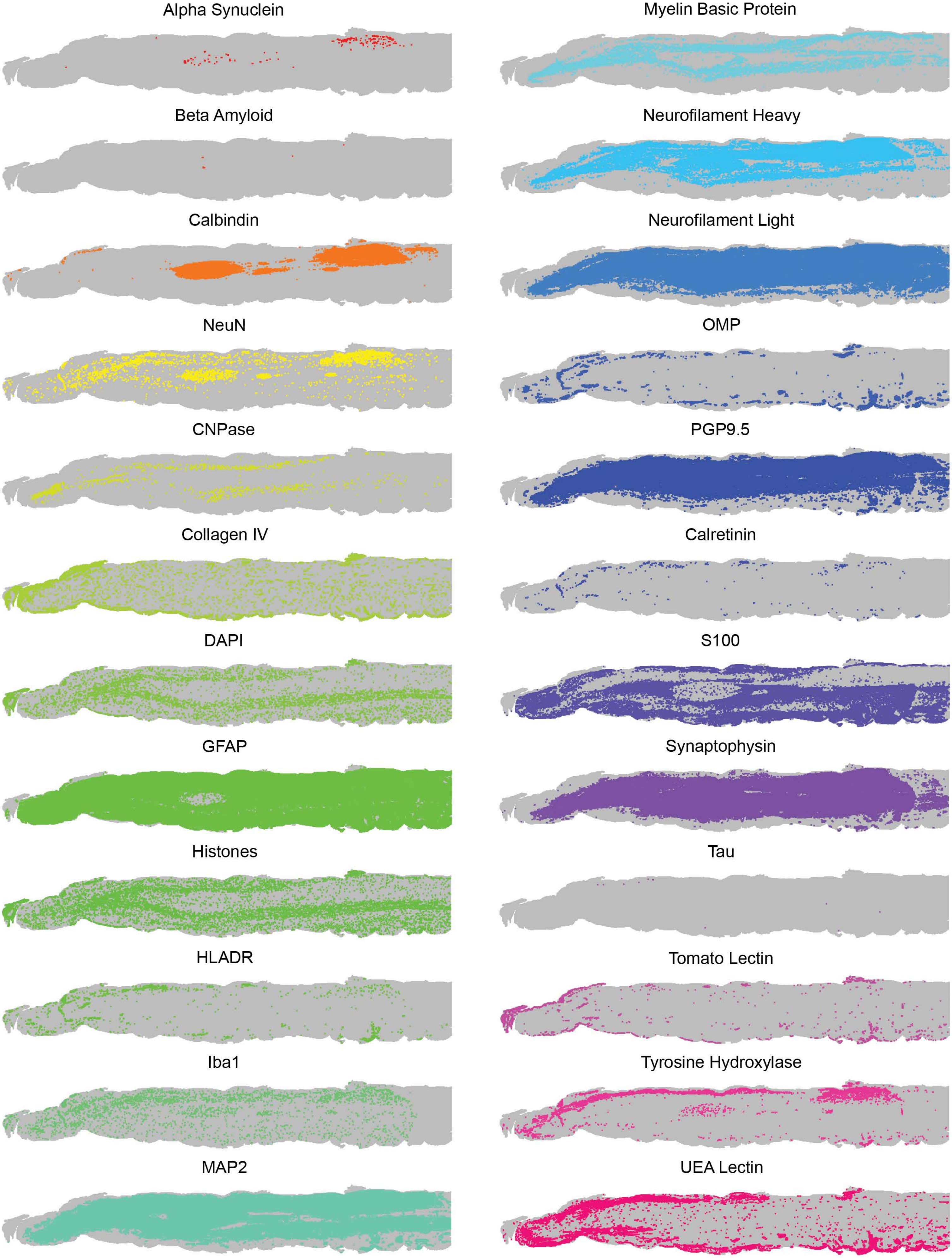
Alpha Synuclein
Myelin Basic Protein
Beta Amyloid
Neurofilament Heavy
Calbindin
Neurofilament Light
NeuN
OMP
CNPase
PGP9.5
Collagen IV
Calretinin
DAPI
S100
GFAP
Synaptophysin
Histones
Tau
HLADR
Tomato Lectin
Iba1
Tyrosine Hydroxylase
MAP2
UEA Lectin

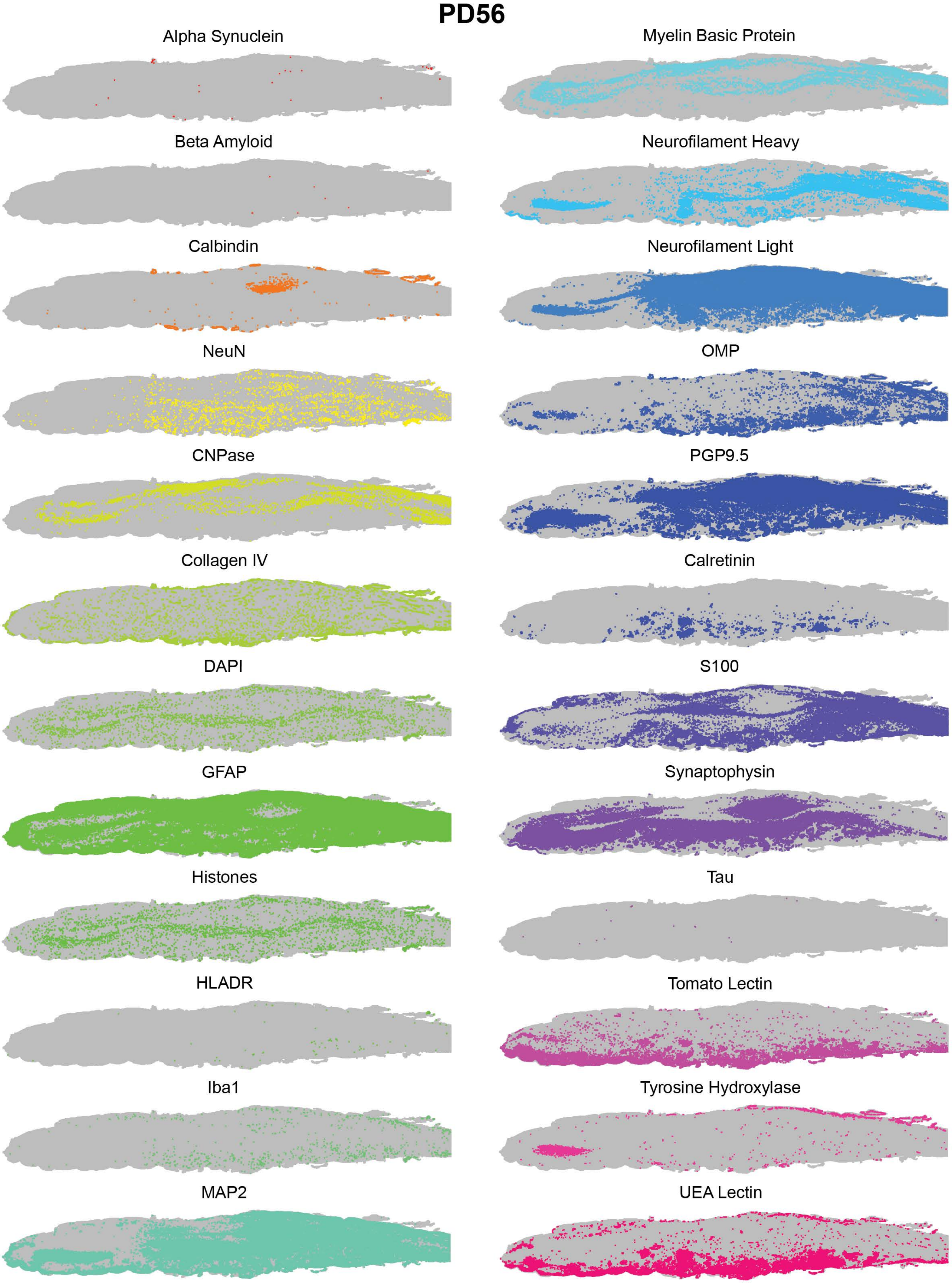
PD56
Alpha Synuclein
Myelin Basic Protein
Beta Amyloid
Neurofilament Heavy
Calbindin
Neurofilament Light
NeuN
OMP
CNPase
PGP9.5
Collagen IV
Calretinin
DAPI
S100
GFAP
Synaptophysin
Histones
Tau
HLADR
Tomato Lectin
Iba1
Tyrosine Hydroxylase
MAP2
UEA Lectin

PD58

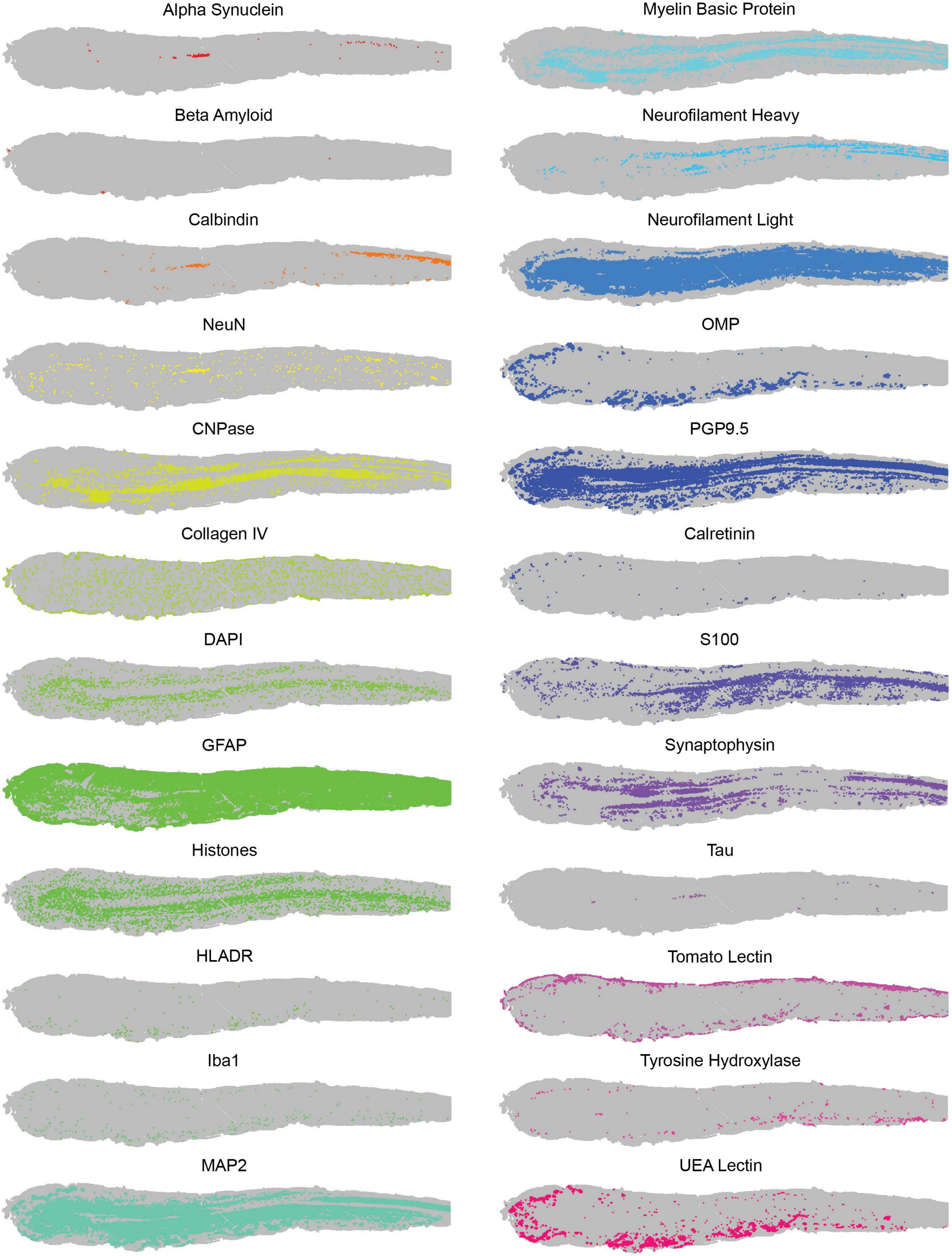
Alpha Synuclein
Myelin Basic Protein
Beta Amyloid
Neurofilament Heavy
Calbindin
Neurofilament Light
NeuN
OMP
CNPase
PGP9.5
Collagen IV
Calretinin
DAPI
S100
GFAP
Synaptophysin
Histones
Tau
HLADR
Tomato Lectin
Iba1
Tyrosine Hydroxylase
MAP2
UEA Lectin

PD77

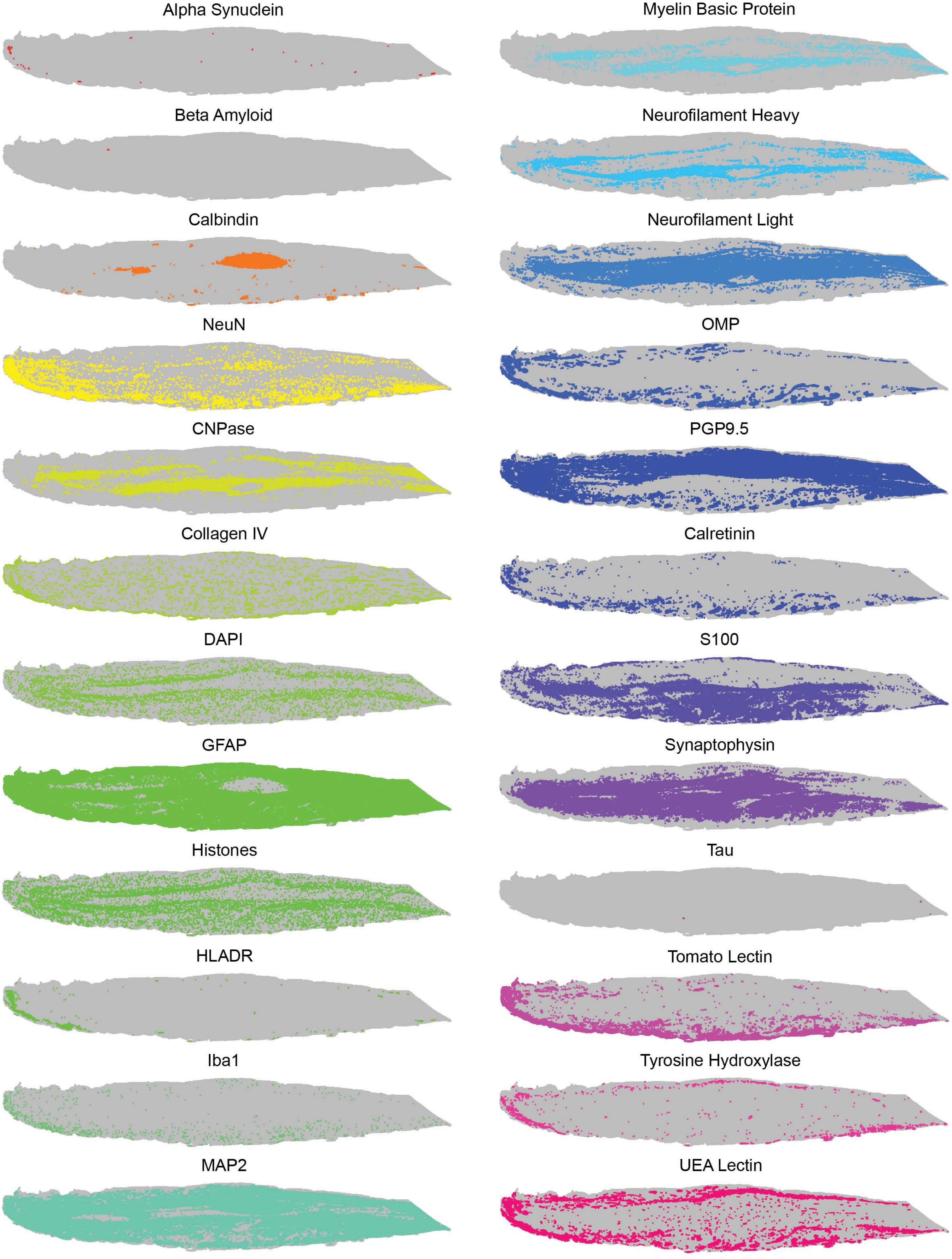

Alpha Synuclein
Myelin Basic Protein
Beta Amyloid
Neurofilament Heavy
Calbindin
Neurofilament Light
NeuN
OMP
CNPase
PGP9.5
Collagen IV
Calretinin
DAPI
S100
GFAP
Synaptophysin
Histones
Tau
HLADR
Tomato Lectin
Iba1
Tyrosine Hydroxylase
MAP2
UEA Lectin

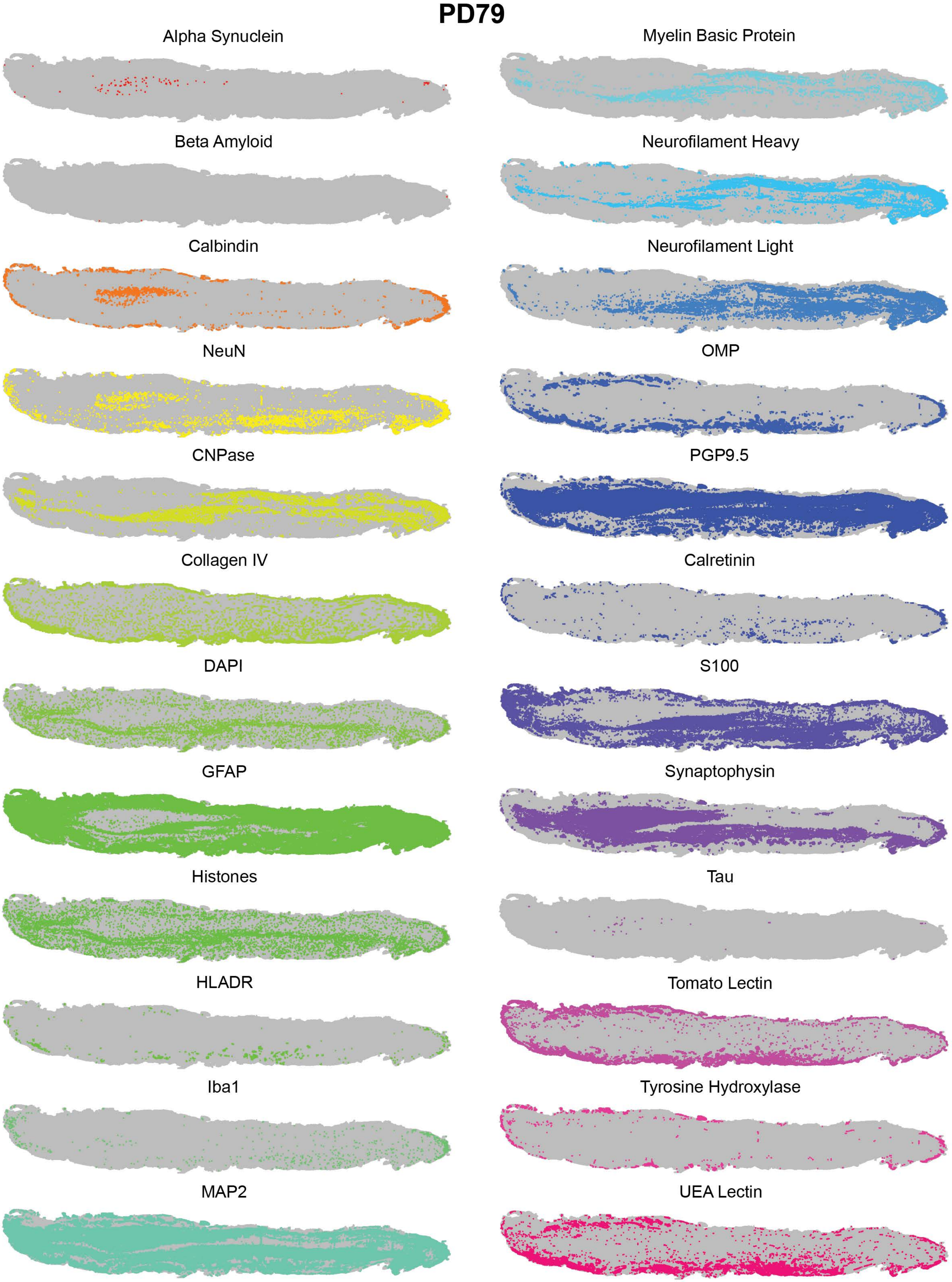
PD79
Alpha Synuclein
Myelin Basic Protein
Beta Amyloid
Neurofilament Heavy
Calbindin
Neurofilament Light
NeuN
OMP
CNPase
PGP9.5
Collagen IV
Calretinin
DAPI
S100
GFAP
Synaptophysin
Histones
Tau
HLADR
Tomato Lectin
Iba1
Tyrosine Hydroxylase
MAP2
UEA Lectin

Supplementary Figure 7. Slide plots of thresholded markers per section. To account for differences in absolute fluorescence intensity between sections, the bins within each section were thresholded for each marker based on Poisson distribution Z-score > 0.05 across that section.

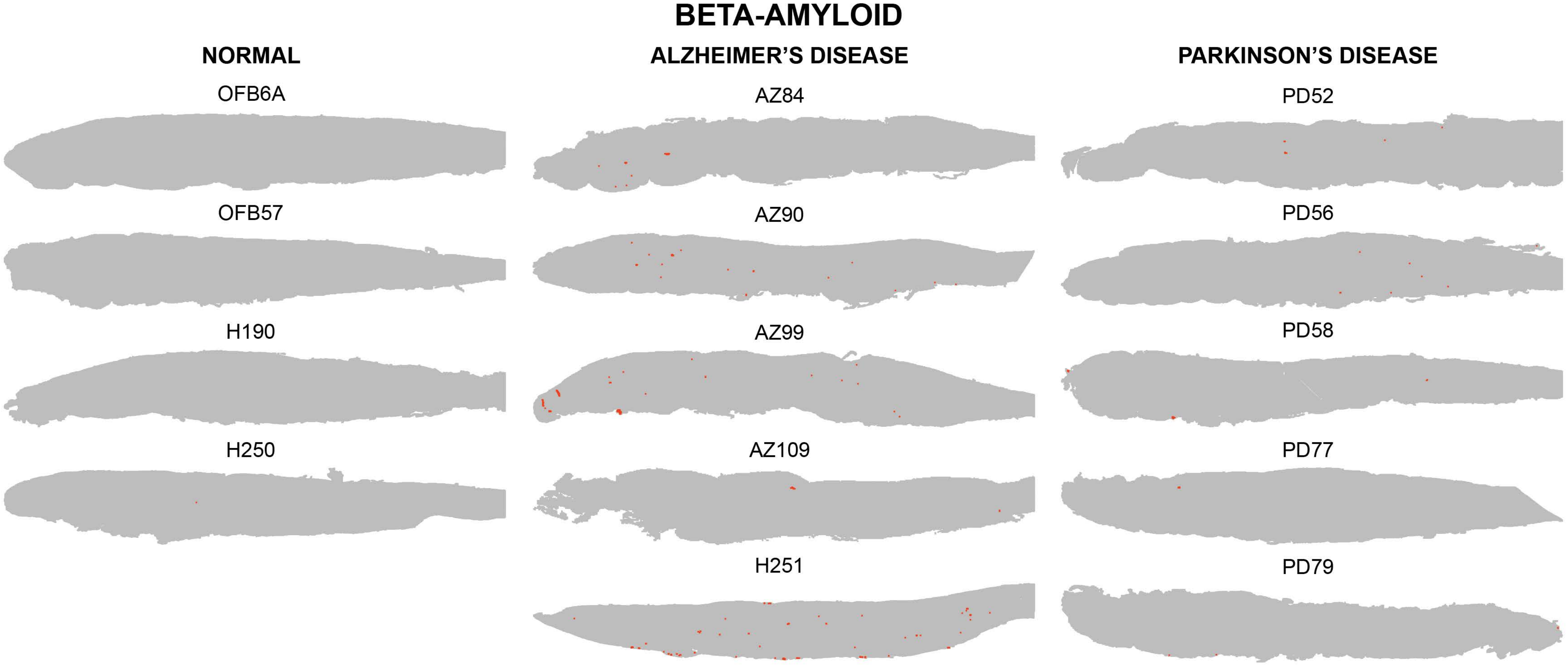
BETA-AMYLOID
NORMAL
ALZHEIMER'S DISEASE
PARKINSON'S DISEASE
OFB6A
OFB57
H190
H250
AZ84
AZ90
AZ99
AZ109
H251
PD52
PD56
PD58
PD77
PD79

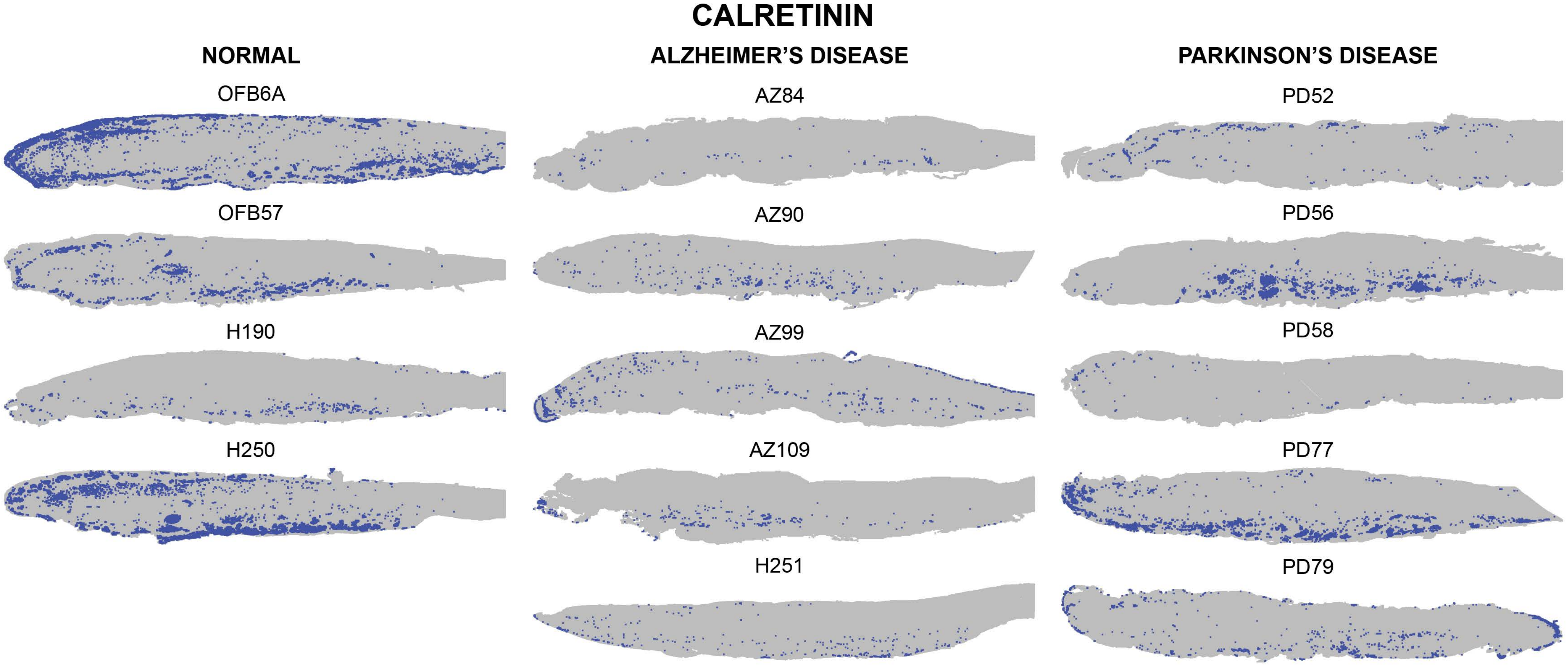
CALRETININ
NORMAL
ALZHEIMER'S DISEASE
PARKINSON'S DISEASE
OFB6A
AZ84
PD52
OFB57
AZ90
PD56
H190
AZ99
PD58
H250
AZ109
PD77
H251
PD79

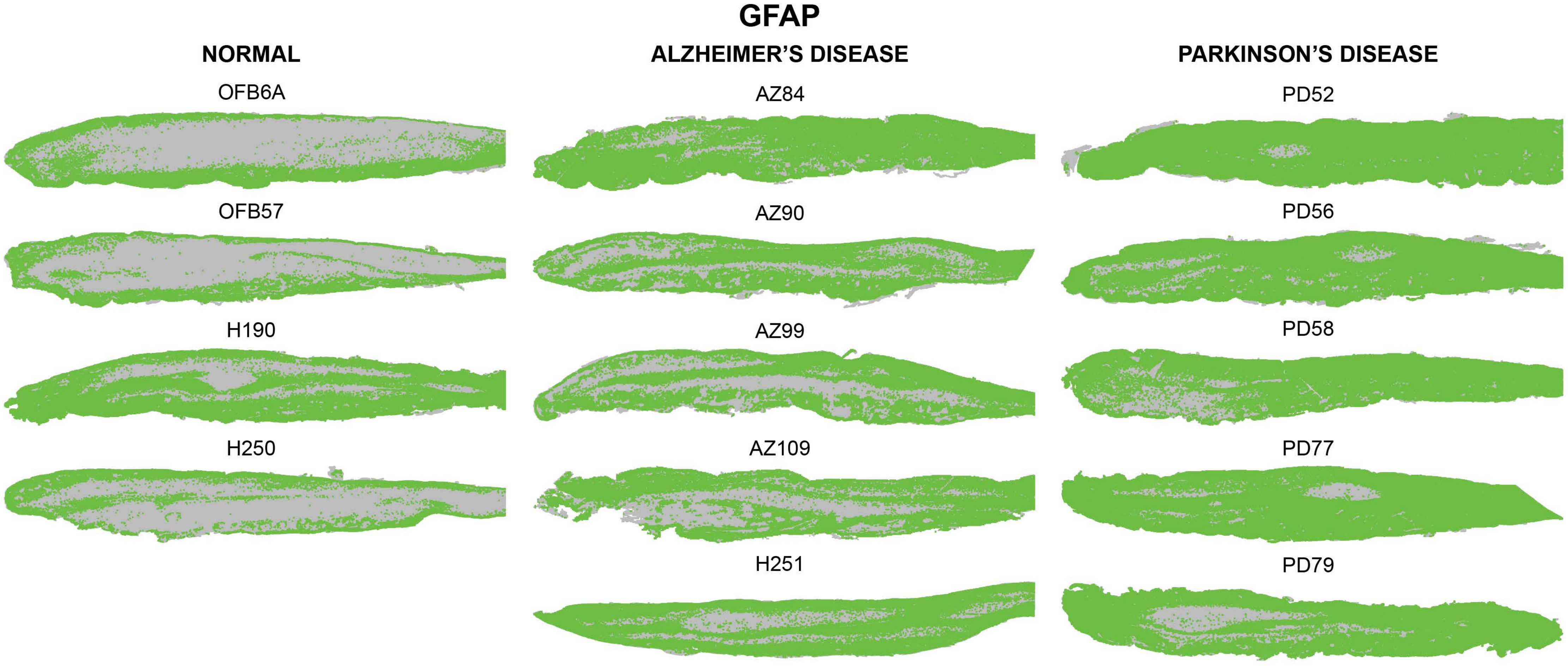
GFAP
NORMAL
ALZHEIMER'S DISEASE
PARKINSON'S DISEASE
OFB6A
AZ84
PD52
OFB57
AZ90
PD56
H190
AZ99
PD58
H250
AZ109
PD77
H251
PD79

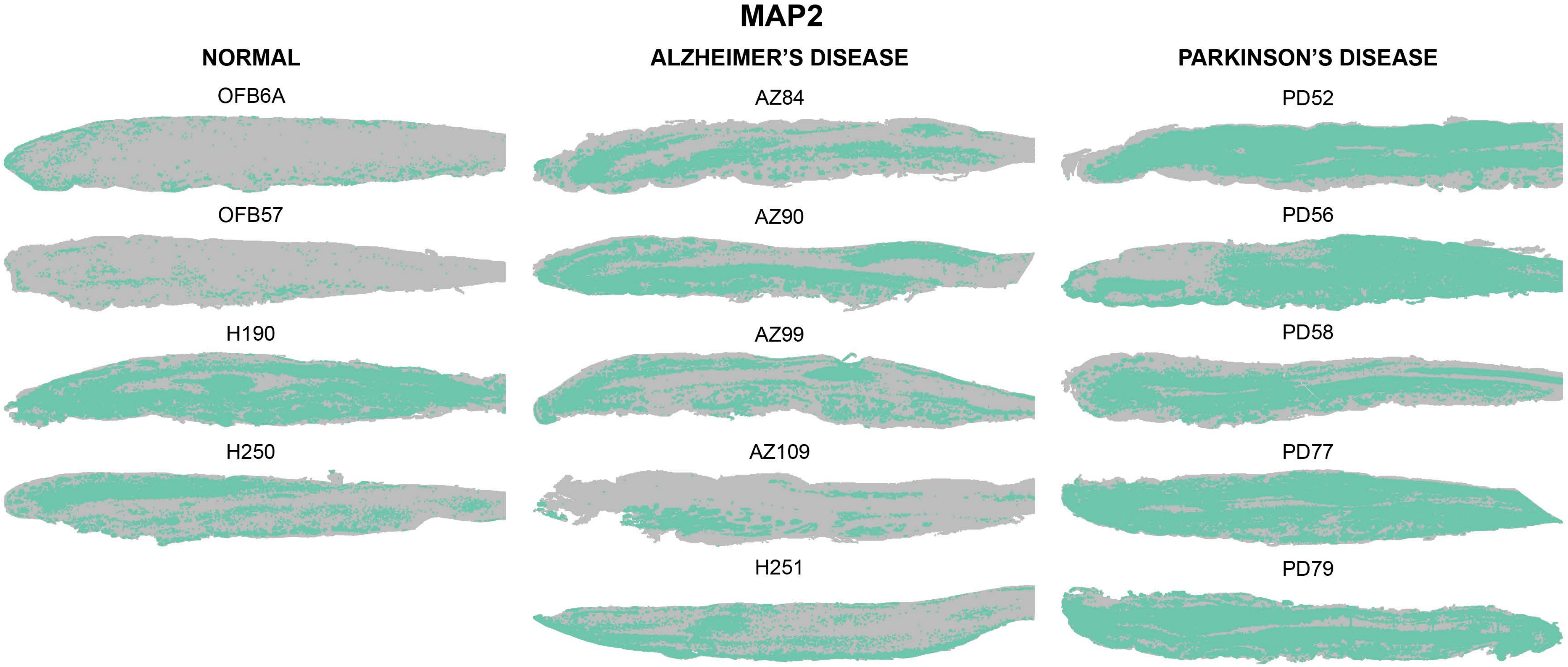
MAP2
NORMAL
ALZHEIMER'S DISEASE
PARKINSON'S DISEASE
OFB6A
OFB57
H190
H250
AZ84
AZ90
AZ99
AZ109
H251
PD52
PD56
PD58
PD77
PD79

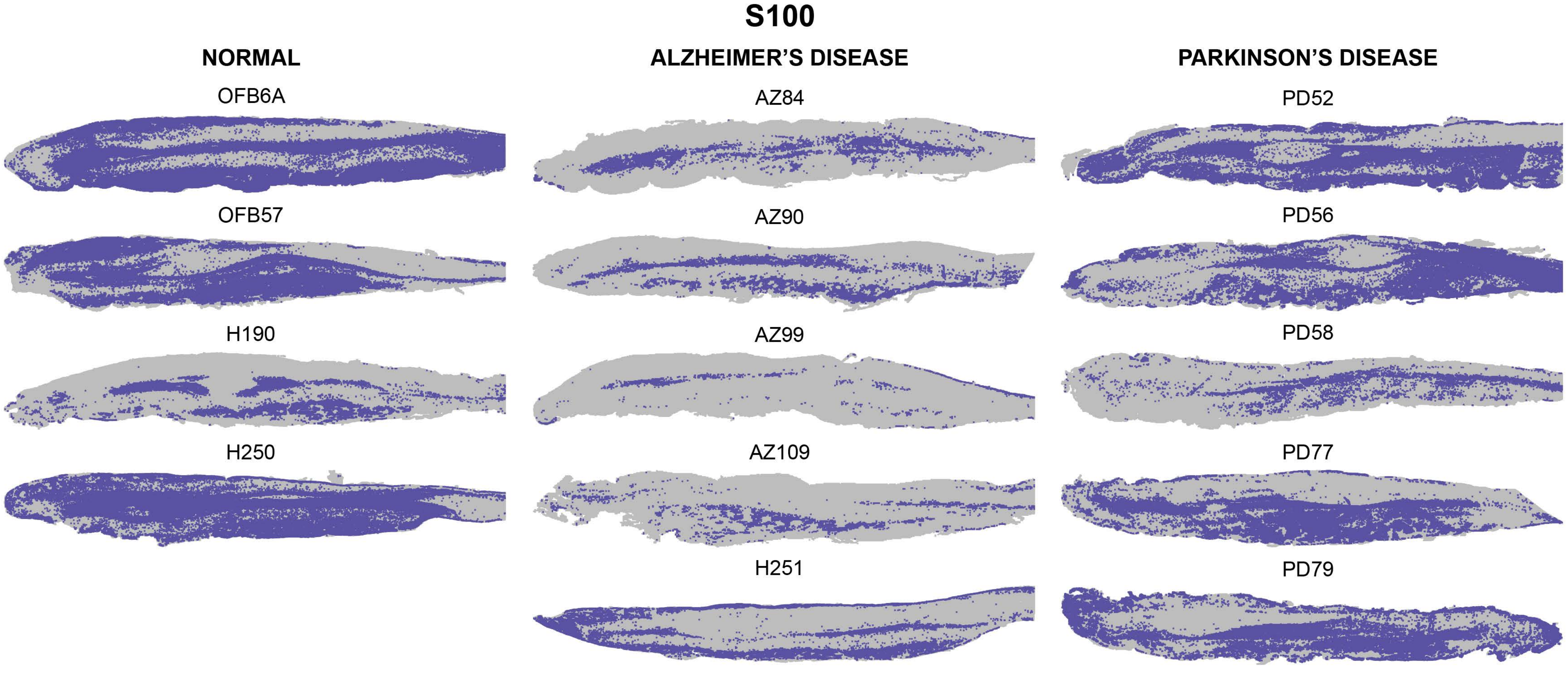
S100
NORMAL
ALZHEIMER'S DISEASE
PARKINSON'S DISEASE
OFB6A
OFB57
H190
H250
AZ84
AZ90
AZ99
AZ109
H251
PD52
PD56
PD58
PD77
PD79

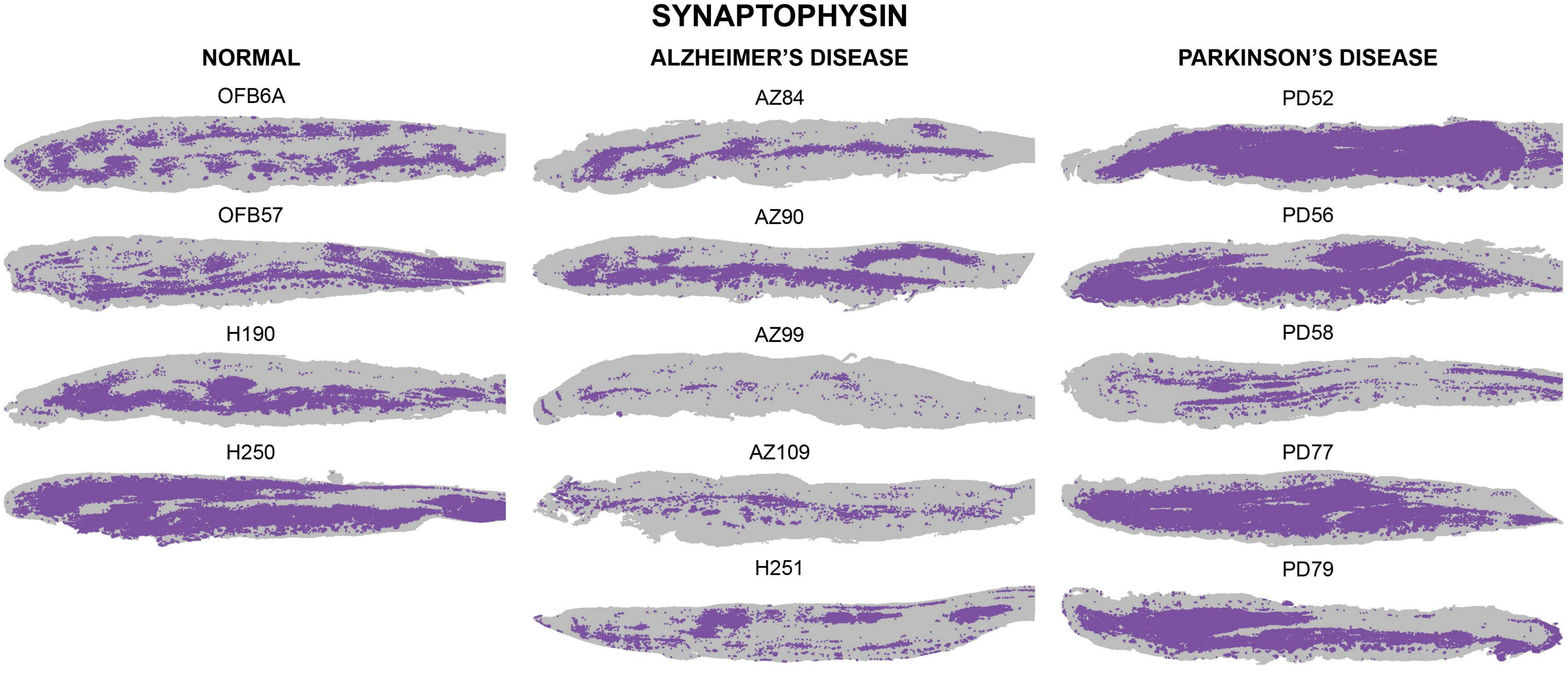
SYNAPTOPHYSIN
NORMAL
ALZHEIMER'S DISEASE
PARKINSON'S DISEASE
OFB6A
AZ84
PD52
OFB57
AZ90
PD56
H190
AZ99
PD58
H250
AZ109
PD77
H251
PD79

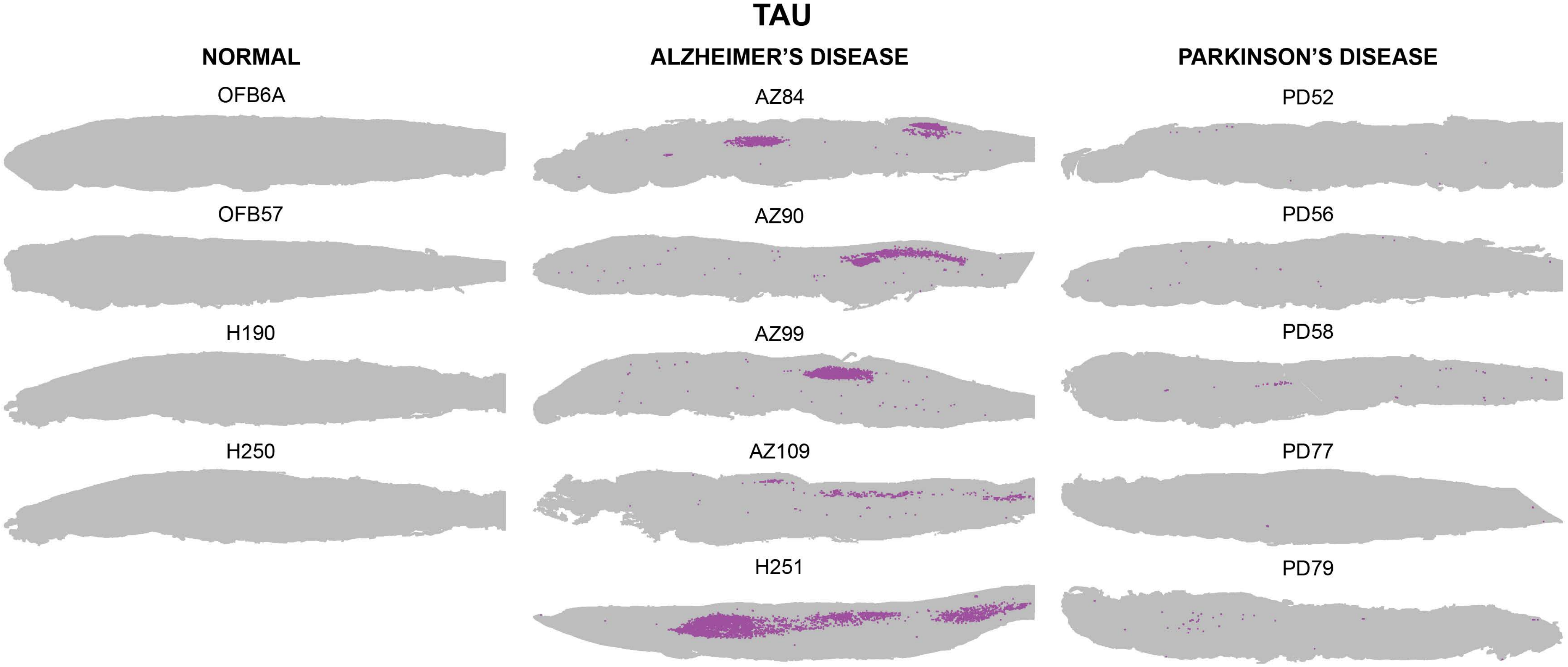
TAU
NORMAL
ALZHEIMER'S DISEASE
PARKINSON'S DISEASE
OFB6A
AZ84
PD52
OFB57
AZ90
PD56
H190
AZ99
PD58
H250
AZ109
PD77
H251
PD79

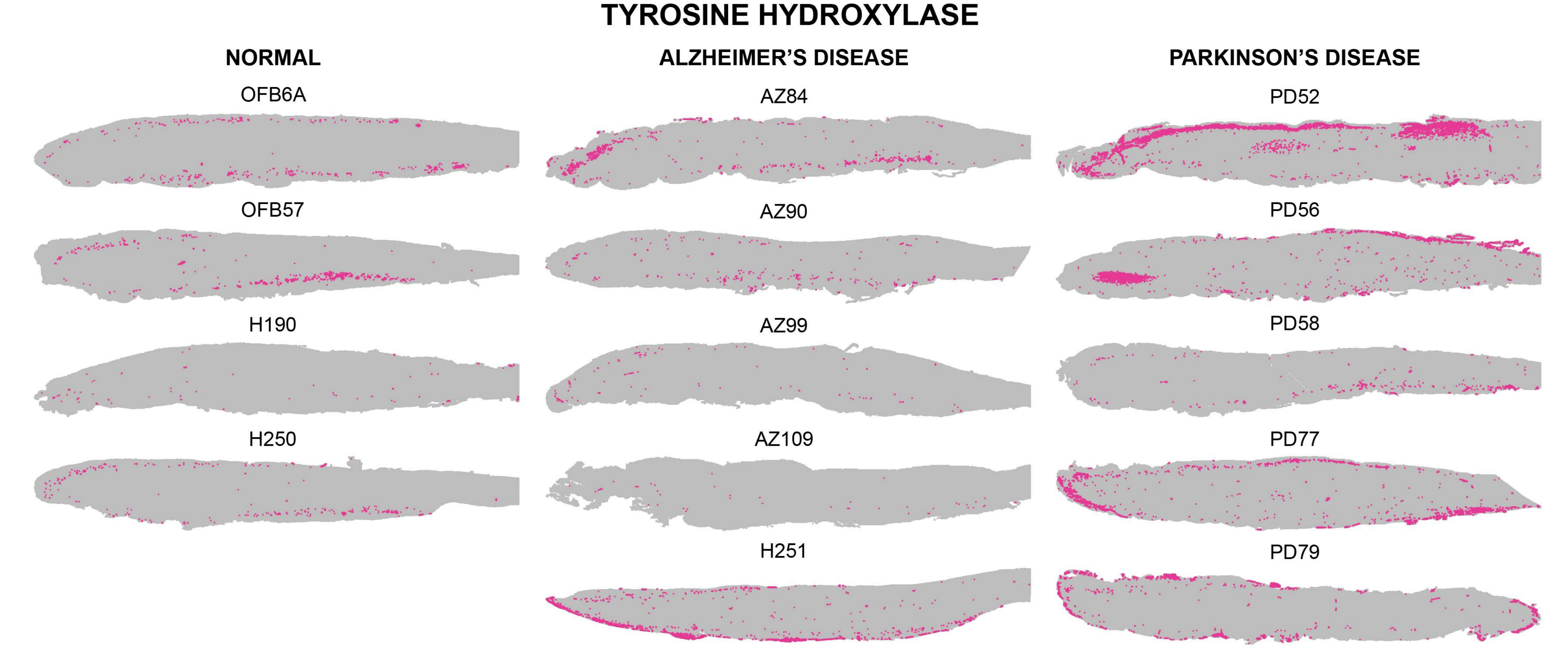
TYROSINE HYDROXYLASE
NORMAL
ALZHEIMER'S DISEASE
PARKINSON'S DISEASE
OFB6A
OFB57
H190
H250
AZ84
AZ90
AZ99
AZ109
H251
PD52
PD56
PD58
PD77
PD79

Supplementary Figure 8. Slide plots of differentially expressed markers per section. The markers beta-amyloid, calretinin, GFAP, MAP2, S100, synaptophysin, tau and tyrosine hydroxylase were determined to be differentially expressed between disease groups at $P < 0.1$. The slide plots for these markers are summarised by case.
