# Supplementary material for: Lamina-specific immunohistochemical signatures in the olfactory bulb of healthy, Alzheimer’s and Parkinson’s disease patients
Source: Commun Biol. 2022 Jan 24;5:88. doi: 10.1038/s42003-022-03032-5 (PMC8786934; doi:10.1038/s42003-022-03032-5)
Supplement: Supplementary file 3 — Description of Additional Supplementary Files [file 42003_2022_3032_MOESM3_ESM.pdf]

Description of Additional Supplementary Files

File name: Supplementary Data 1
Description: Ratio of positive bins : total bins per marker, per image and statistical analysis
