# Supplementary material for: Lamina-specific immunohistochemical signatures in the olfactory bulb of healthy, Alzheimer’s and Parkinson’s disease patients
Source: Commun Biol. 2022 Jan 24;5:88. doi: 10.1038/s42003-022-03032-5 (PMC8786934; doi:10.1038/s42003-022-03032-5)
Supplement: Supplementary file 5 — Reporting Summary [file 42003_2022_3032_MOESM5_ESM.pdf]

nature portfolio

Corresponding author(s): Dr Helen Murray & Dr Dragan Maric

Last updated by author(s): Dec 7, 2021

Reporting Summary

Nature Portfolio wishes to improve the reproducibility of the work that we publish. This form provides structure for consistency and transparency in reporting. For further information on Nature Portfolio policies, see our Editorial Policies and the Editorial Policy Checklist.

Statistics

For all statistical analyses, confirm that the following items are present in the figure legend, table legend, main text, or Methods section.

n/a	Confirmed	
☐	☒	The exact sample size (*n*) for each experimental group/condition, given as a discrete number and unit of measurement
☐	☒	A statement on whether measurements were taken from distinct samples or whether the same sample was measured repeatedly
☐	☒	The statistical test(s) used AND whether they are one- or two-sided<br>*Only common tests should be described solely by name; describe more complex techniques in the Methods section.*
☒	☐	A description of all covariates tested
☐	☒	A description of any assumptions or corrections, such as tests of normality and adjustment for multiple comparisons
☐	☒	A full description of the statistical parameters including central tendency (e.g. means) or other basic estimates (e.g. regression coefficient) AND variation (e.g. standard deviation) or associated estimates of uncertainty (e.g. confidence intervals)
☐	☒	For null hypothesis testing, the test statistic (e.g. *F*, *t*, *r*) with confidence intervals, effect sizes, degrees of freedom and *P* value noted<br>*Give P values as exact values whenever suitable.*
☒	☐	For Bayesian analysis, information on the choice of priors and Markov chain Monte Carlo settings
☒	☐	For hierarchical and complex designs, identification of the appropriate level for tests and full reporting of outcomes
☒	☐	Estimates of effect sizes (e.g. Cohen's *d*, Pearson's *r*), indicating how they were calculated

Our web collection on statistics for biologists contains articles on many of the points above.

Software and code

Policy information about availability of computer code

Data collection	We used commercially available Zeiss ZEN 2 software for image acquisition.
Data analysis	Adobe Photoshop was used for image alignment. We used R (https://www.r-project.org/) and the Seurat library (https://rdocumentation.org/packages/Seurat/versions/3.1.4) for image analysis.

For manuscripts utilizing custom algorithms or software that are central to the research but not yet described in published literature, software must be made available to editors and reviewers. We strongly encourage code deposition in a community repository (e.g. GitHub). See the Nature Portfolio guidelines for submitting code & software for further information.

Data

Policy information about availability of data

All manuscripts must include a data availability statement. This statement should provide the following information, where applicable:

- Accession codes, unique identifiers, or web links for publicly available datasets
- A description of any restrictions on data availability
- For clinical datasets or third party data, please ensure that the statement adheres to our policy

The data that support the findings of this study are available from the corresponding author upon reasonable request. The original images used for analysis are publicly hosted at https://doi.org/10.17608/k6.auckland.14920233. The original code used for the analysis is publicly hosted at https://doi.org/10.17608/k6.auckland.17132603.v1.

Field-specific reporting

Please select the one below that is the best fit for your research. If you are not sure, read the appropriate sections before making your selection.

- [x] Life sciences
- [ ] Behavioural & social sciences
- [ ] Ecological, evolutionary & environmental sciences

For a reference copy of the document with all sections, see nature.com/documents/nr-reporting-summary-flat.pdf

Life sciences study design

All studies must disclose on these points even when the disclosure is negative.

Sample size	The data consists of 83-channel stitched images scanned from 7-micron thick human olfactory bulb sections from 5 Alzheimer's disease, 5 Parkinson's disease and 4 neurologically normal subjects with the size of each raw image for each acquired channel approximately 2.5GB. The quantification analysis on individual sections resulted in detection of approximately 100,000 bins per section with 28-plex phenotypic information. We considered the provided sample size to be adequate for the purpose of demonstrating the effectiveness of our imaging and analysis methods.
Data exclusions	Across the labelling rounds we tested antibodies from different manufacturers to the same antigen and some antibodies were repeated across different labelling rounds. The 25 markers selected (from a total of 83 labelled) for the spatial protein analysis were those that showed the most robust and reproducible labelling across all sections.
Replication	The antibody and labelling conditions have been stated in sufficient detail to reproduce these findings and all antibodies have been previously validated (Maric et al., 2021. Nature Communications). From the quantitative analysis point, both the data and codes are made publicly available to anyone for reproduction.
Randomization	Allocation of cases into disease groups was not random. The AD, PD or normal diagnosis was determined by an independent pathologist who carried out a neuropathology assessment of the brains for each case (Table 2). The four neurologically normal cases had no history of neurological abnormalities, and no other neuropathology was noted. The five AD cases had a clinical history of dementia, and the clinical AD diagnosis was confirmed by independent pathological assessment. The presence of tau pathology in the OB was previously verified. The five PD cases had a clinical diagnosis of PD, which was confirmed by independent pathological assessment. The presence of alpha-synuclein pathology in the OB was previously verified.
Blinding	The spatial protein analysis was carried out in an automated unbiased manner, such that the investigator does not input the disease group for each tissue section into the analysis.

Reporting for specific materials, systems and methods

We require information from authors about some types of materials, experimental systems and methods used in many studies. Here, indicate whether each material, system or method listed is relevant to your study. If you are not sure if a list item applies to your research, read the appropriate section before selecting a response.

Materials & experimental systems

n/a	Involved in the study	
☐	☒	Antibodies
☒	☐	Eukaryotic cell lines
☒	☐	Palaeontology and archaeology
☒	☐	Animals and other organisms
☐	☒	Human research participants
☒	☐	Clinical data
☒	☐	Dual use research of concern

Methods

n/a	Involved in the study	
☒	☐	ChIP-seq
☒	☐	Flow cytometry
☒	☐	MRI-based neuroimaging

Antibodies

Antibodies used	All antibody information is provided in supplementary table 1
Validation	All antibodies used in this study have been previously validated in the literature and the validation information for each antibody is available from the source vendors where the antibodies were purchased. The relevant vendor information for each antibody is provided in supplementary table 1

Human research participants

Policy information about studies involving human research participants

Population characteristics	All subject characteristics including diagnosis, age, sex, post-mortem delay and pathology scores have been provided in the methods section
Recruitment	Human olfactory bulbs were obtained from the Neurological Foundation Human Brain Bank and the Human Anatomy Laboratory within the Department of Anatomy and Medical Imaging at the University of Auckland, New Zealand. The sample was selected on the basis of tissue availability for each disease group.
Ethics oversight	The ethical approval for tissue collection was provided by the New Zealand Health and Disability Ethics Committee (ref 14/NTA/208 and the University of Auckland Human Participant Ethics Committee (ref 011654)

Note that full information on the approval of the study protocol must also be provided in the manuscript.
